# Understanding a mass in the paraspinal region: an anatomical approach

**DOI:** 10.1186/s13244-023-01462-1

**Published:** 2023-07-19

**Authors:** Maud Creze, Jessica Ghaouche, Gilles Missenard, Thierry Lazure, Guillaume Cluzel, Matthieu Devilder, Sylvain Briand, Marc Soubeyrand, Olivier Meyrignac, Robert-Yves Carlier, Charles Court, Charlie Bouthors

**Affiliations:** 1 Department of Radiology, Assistance Publique des Hôpitaux de Paris, GH Université Paris- Saclay, DMU Smart Imaging, Bicêtre Teaching Hospital, Le Kremlin-Bicêtre, France; 2 BioMaps, Université Paris-Saclay, Hôpital Kremlin-Bicêtre, 78 rue du Général Leclerc, 94270 Le Kremlin-Bicêtre, France; 3 Department of Orthopedic Surgery, Assistance Publique des Hôpitaux de Paris, GH Université Paris-Saclay, DMU de Chirurgie Traumatologie Orthopédique-Chirurgie Plastique- Reconstruction, Bicêtre Teaching Hospital, Le Kremlin-Bicêtre, France; 4 Department of Pathology, Assistance Publique des Hôpitaux de Paris, GH Université Paris-Saclay, DMU Smart Imaging, Bicêtre hospital, Le Kremlin Bicêtre, France; 5 Clinique Saint Jean L’Ermitage, Santépôle, Melun, France; 6 Department of Radiology, Assistance Publique des Hôpitaux de Paris, GH Université Paris- Saclay, DMU Smart Imaging, Garches Teaching Hospital, Le Kremlin-Bicêtre, France

**Keywords:** Anatomy, Imaging, Paraspinal muscle, Soft tissue neoplasm, Spinal neoplasm

## Abstract

The paraspinal region encompasses all tissues around the spine. The regional anatomy is complex and includes the paraspinal muscles, spinal nerves, sympathetic chains, Batson’s venous plexus and a rich arterial network. A wide variety of pathologies can occur in the paraspinal region, originating either from paraspinal soft tissues or the vertebral column. The most common paraspinal benign neoplasms include lipomas, fibroblastic tumours and benign peripheral nerve sheath tumours. Tumour-like masses such as haematomas, extramedullary haematopoiesis or abscesses should be considered in patients with suggestive medical histories. Malignant neoplasms are less frequent than benign processes and include liposarcomas and undifferentiated sarcomas. Secondary and primary spinal tumours may present as midline expansile soft tissue masses invading the adjacent paraspinal region. Knowledge of the anatomy of the paraspinal region is of major importance since it allows understanding of the complex locoregional tumour spread that can occur via many adipose corridors, haematogenous pathways and direct contact. Paraspinal tumours can extend into other anatomical regions, such as the retroperitoneum, pleura, posterior mediastinum, intercostal space or extradural neural axis compartment. Imaging plays a crucial role in formulating a hypothesis regarding the aetiology of the mass and tumour staging, which informs preoperative planning. Understanding the complex relationship between the different elements and the imaging features of common paraspinal masses is fundamental to achieving a correct diagnosis and adequate patient management. This review gives an overview of the anatomy of the paraspinal region and describes imaging features of the main tumours and tumour-like lesions that occur in the region.

**Supplementary Information:**

The online version contains supplementary material available at 10.1186/s13244-023-01462-1.

## Key points

1. Paraspinal tumours can invade the epidural/intercostal spaces, mediastinum, pleura and retroperitoneum.
2. Paraspinal tumours originate either from the spine or the paraspinal soft tissues.
3. Paraspinal soft tissue tumours can invade the adjacent vertebra or rib.
4. MRI is needed to assess the anatomical location of paraspinal lesions.
5. Image-guided biopsy is required to determine the histological nature of the mass.

**Supplementary Information:**

The online version contains supplementary material available at 10.1186/s13244-023-01462-1.

## Background

Tumours of the paraspinal region are rare entities that develop at the intersection of many anatomical structures. This region is complex, and because of this, correct and meaningful analysis of paraspinal lesions is challenging and requires skill based on a true understanding of the anatomy of paraspinal tissues.

Broadly, the paraspinal region encompasses all soft tissues located around the spine, between the parietal fascia ventrally and the paraspinal muscle aponeurosis dorsally. The particular location of the paraspinal region, which is in contact with various components of the spine, explains the great diversity of tumours that can occur. Accordingly, tumours of the paraspinal soft tissues originate from either the spine (and so are categorised as bone tumours) or the paraspinal soft tissues themselves (categorised as soft tissue tumours). Certainly, the origin and the histology of these tumours are very different, but the anatomical and radiological boundaries between the spine and paraspinal soft tissues are thin and are often crossed. This explains why some spinal tumours invade the paraspinal region more readily than the bone itself, and reciprocally, soft tissue tumours, even benign ones, commonly invade the vertebrae. Some tumour mimics can also occur in the paraspinal region.

Whether in analysis of soft tissue conditions or in characterisation of spinal lesions, the paraspinal region is the ‘poor relative’. Reviews about spinal tumours usually cover only the spinal lesion and do not describe any paraspinal extension. Meanwhile, tumours of the paraspinal soft tissues are usually studied together with other tumours of the trunk, including retroperitoneal, abdominal wall or chest wall tumours, and studies typically do not focus on paraspinal masses. However, because of their intimate relationship with critical structures and bone, paraspinal masses should be analysed as a separate entity.

Anatomically, the paraspinal region is not enclosed; it communicates with the extradural neural axis compartment via the intervertebral foramina and with the intercostal spaces. It has a close anatomical relationship with the posterior mediastinum, pleura and retroperitoneum. The rich vascular and neural paraspinal network and the many adipose corridors help to explain how a paraspinal tumour process can invade adjacent compartments in various ways: via locoregional haematogenous dissemination, perineural spread and direct contact. Complex anatomical relationships with critical structures explain the surgical difficulties around achieving complete tumour resection and the need for extensive radiological expertise for preoperative planning.

This educational review aims to precisely describe the soft tissues around the spine and exhibit features of tumours and differential diagnoses. It first provides an anatomical description of the paraspinal region, setting out the key points necessary for the understanding and description of paraspinal lesions. We put an emphasis on the pathways involved in tumour spreading. It then focuses on the characteristics of some paraspinal tumours that should lead radiologists towards suggesting a specific diagnosis. Finally, it gives the main principles of the surgical treatment of these lesions. This educational review is not intended to be an exhaustive review of spinal tumours or soft tissue masses but rather to give radiologists some keys to unlock this complex anatomical region and its issues.

The article will not cover pathologies originating from the skin, posterior mediastinum, extrapleural space, retroperitoneum or oropharyngeal tract.

## Anatomy

### Muscles

Muscle tissue is the most predominant tissue of the paraspinal region. According to their location in relation to the transverse processes (anterior or posterior), paraspinal muscles are classified, in vertebrates, as either ventral hypaxial or dorsal epaxial muscles. Muscle distribution, number and size vary according to the spinal level, relating to the specific functional role of the cervical, thoracic and lumbar regions [1, 2].

**Fig. 1:**
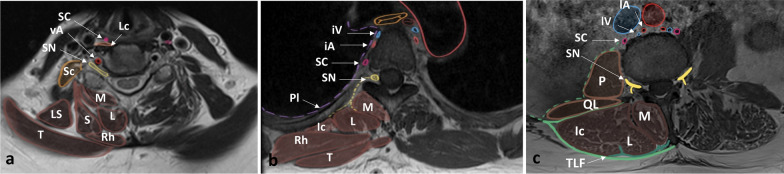
*Anatomy of the paraspinal region* at C6 level (**a**), at T4 level (**b**) and at L3 level (**c**) on axial T1WI. T, trapezius; S, splenius; Rh, rhomboid; iA, intercostal artery; Iv, intercostal vein; M, multifidus; L, longissimus; Ic, iliocostalis; Sc, sympathetic chain; LS, levator scapulae; Sc, scalenus; vA, vertebral artery; Pl, pleura; SN, spinal nerve; P, psoas; QL, quadratus lumborum; TLF, thoracolumbar fascia; and Lc, longus colli

**Fig. 2:**
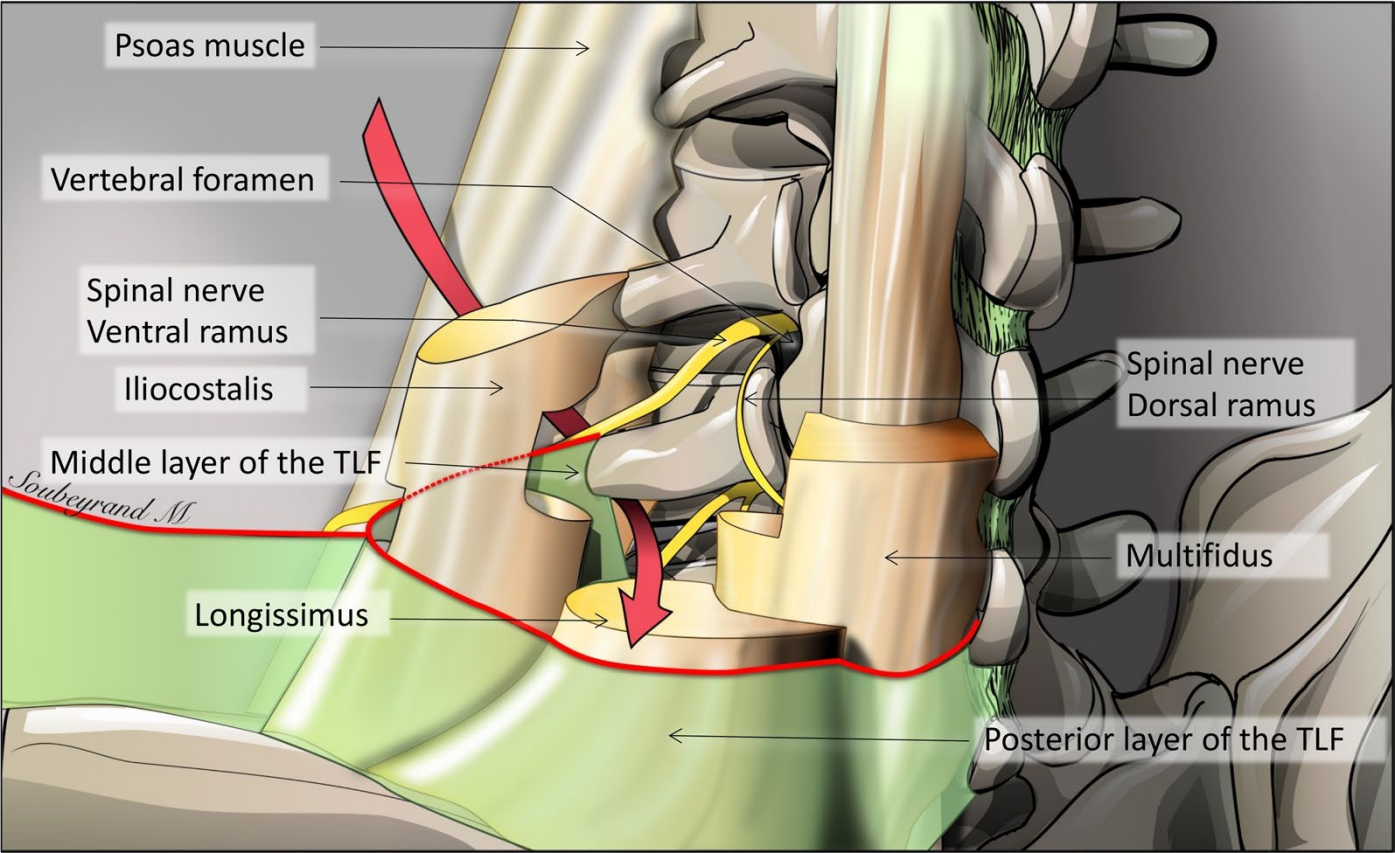
*Posterior view of the lumbar paraspinal region*. TLF: thoracolumbar fascia. The red arrow shows the communication between epaxial and hypaxial regions

**Fig. 3:**
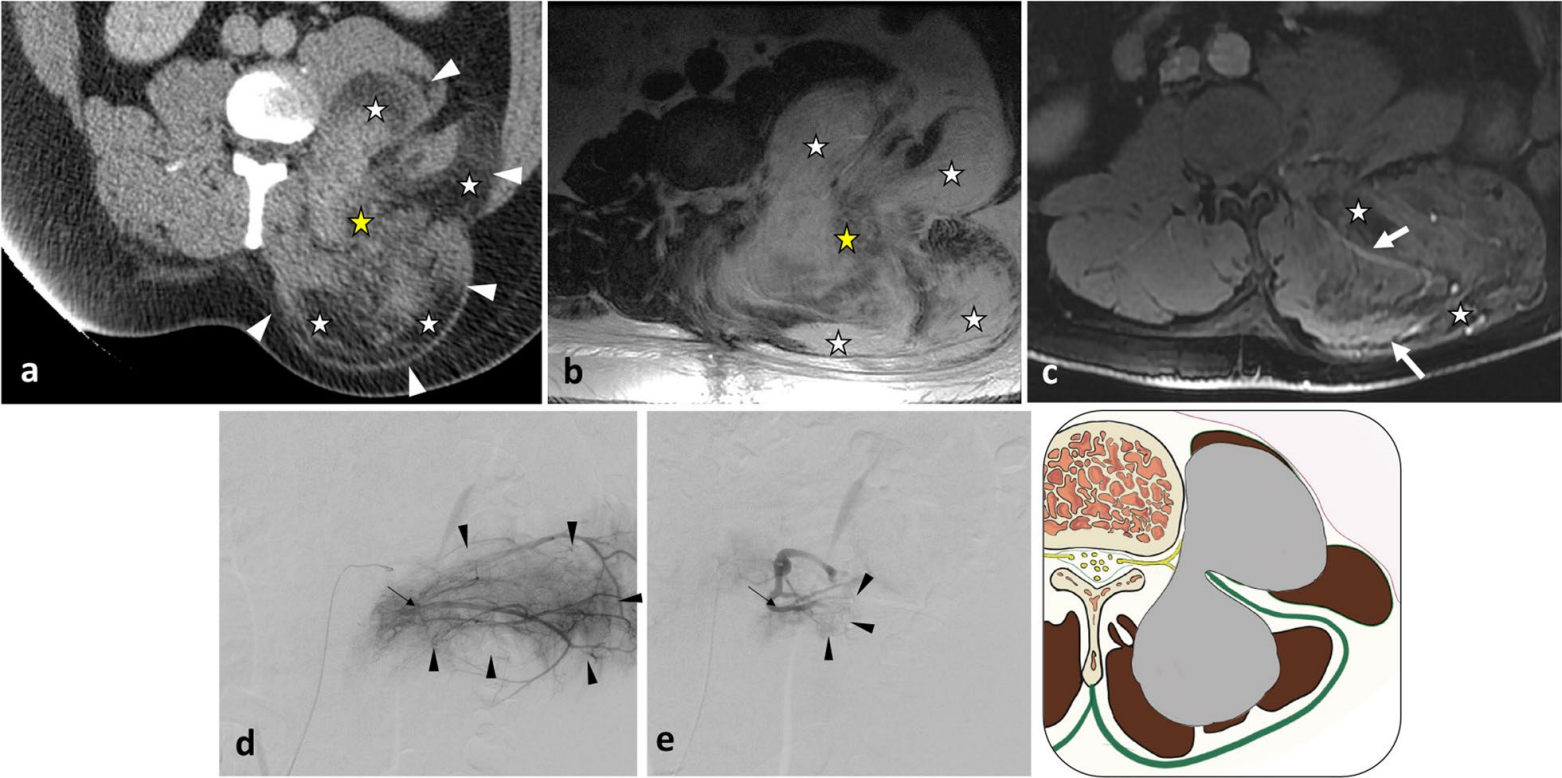
*Liposarcoma* of the left lumbar paraspinal muscles in a 57-year-old male with a bump in the back and mild left iliac fossa pain. Contrast-enhanced axial CT scan reconstruction (**a**) and axial T1WI (**b**) show intramuscular lipomatous tumour invading both the hypaxial and epaxial muscles. Fat content (white stars) presents low attenuation on CT, high signal on T1WI and saturates on fat-saturated T1WI (c). The mass demonstrates a bilobed shape with an epicentre located between the epaxial and hypaxial regions (yellow stars). Axial contrast-enhanced fat-saturated T1WI (**c**) shows muscle and fascia enhancement (arrows), a key driving feature for liposarcoma. Digital subtraction angiogram (**d**) of the left 2^nd^ lumbar artery (arrow) shows hypervascularisation of the tumour (arrowheads). After embolisation (**e**), hypervascularisation decreased (arrowheads)

Hypaxial muscles are represented by *longus colli* muscles at the cervical level and by *psoas* and *quadratus lumborum* at the lumbar level [3]. There is no hypaxial muscle at the thoracic level, leaving the posterior mediastinum and pleura vulnerable. Epaxial muscles include both minor segmental muscles (the *rotatores*, *intertransversarii* and *interspinales*) and long major polyarticular muscles. At the cervical level, epaxial muscles are numerous and include the *splenius capitis*, *splenius cervicis*, *semispinalis* and *multifidus*. The *trapezius* and *rhomboids* cover the erector spinae at the cervicothoracic level (Figs. 1, 2). At the thoracolumbar level, epaxial muscles consist mainly of two bulky muscles, the *erector spinae* and the *multifidus* [4, 5]. Since muscle and fat are relatively poor barriers to tumour spreading, the paraspinal region is defined as ‘extracompartmental’ in radiological evaluation of soft tissue staging [6]. As paraspinal muscles are long polyarticular muscles, pathological conditions often spread vertically over several segments along muscle fibres and fasciae resulting in ‘giant’ tumours (Fig. 3). Giant tumours are often lobulated and form internal hernias making a wide resection difficult due to proximity to nerve and vessels.

### Fasciae, aponeuroses and the paraspinal compartment

On its posterior surface, the epaxial region, posterior to the transverse processes, is covered by numerous aponeuroses and fibrous fasciae, which explains why fibroblastic tumours are among the most frequent in the region [7]. The trapezius aponeurosis covers the cervicothoracic epaxial muscles, and the erector spinae aponeurosis and the posterior layer of the *thoracolumbar fascia* (TLF) overlap the thoracolumbar spine (Figs.1, 2). These superficial fasciae constitute a biological barrier, which limits the extension of superficial soft tissue tumours inward.

The epaxial and hypaxial regions communicate at all spinal levels. However, at the lumbar level, the TLF partially compartmentalises the epaxial region [8]. It is made of three layers: anterior, middle and posterior. The posterior layer is a thick fibrous sheath covering epaxial muscles. The middle layer is located ventrally to epaxial muscles and forms an intermuscular septum that separates the epaxial from the hypaxial musculature. The middle layer is attached medially to the tips of the transverse processes of the lumbar vertebrae and merges laterally with the posterior layer. At each lumbar level, between the medial attachment of the middle layer, the epaxial and hypaxial regions communicate. This arrangement explains the bilobed shape of some lumbar paraspinal tumours on either side of the middle layer of the TLF (Fig. 3).

**Fig. 4:**
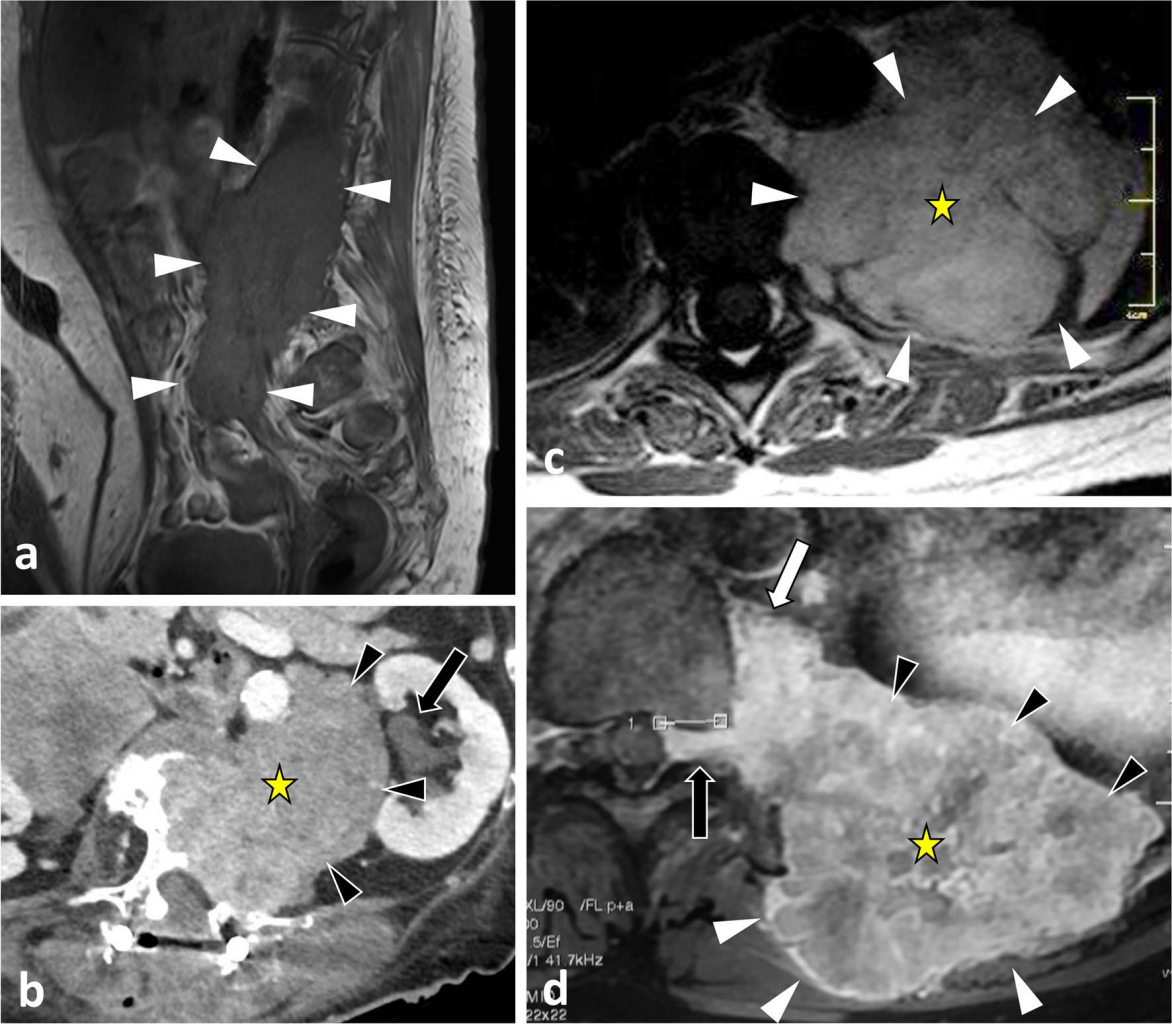
*Longitudinal and transversal extension of three distinct paraspinal tumours. Spinal and paraspinal metastasis* of urothelial carcinoma of the left upper tract in an 83-year-old female (**a**, **b**). Sagittal T1WI **a** shows the longitudinal extension of the tumour within the left psoas muscle from L2 to S1 level. Contrast-enhanced axial CT scan reconstruction (**b**) demonstrates the transversal extension of the mass within the retroperitoneum (arrowheads), causing ureteral stenosis (black arrow). *Undifferentiated sarcoma* of soft tissue in a 37-year-old female (**c**). Contrast-enhanced axial T1WI (**c**) shows pleural extension (arrowhead). *Costal Ewing’s sarcoma* in a 23-year-old male (**d**). Contrast-enhanced axial T1WI (**d**) shows an extension in epaxial muscles (white arrowheads), a pleural and intercostal extension (black arrowheads), a posterior mediastinum extension (white arrow) and an epidural extension via the intervertebral foramen (black arrow). Yellow stars show the epicentre of masses

The hypaxial region is ventrally covered by a parietal fascia which corresponds to the *endoabdominal fascia* (including the *transversalis fascia* [also considered the anterior layer of the TLF] and the *iliaca fascia*) and the *endothoracic fascia* at the lumbar and thoracic levels, respectively [9, 10]. Hypaxial fasciae are thin layers of connective tissues which do not constitute a barrier against ventral paraspinal tumour extension and allow easy invasion of the pleura, posterior mediastinum and retroperitoneum (Fig. 4).

### Nerves

*Spinal nerves* emerge through the intervertebral foramen between adjacent vertebrae, bilaterally (Fig. 2). Immediately below the intervertebral foramen, the spinal nerve divides into two branches, a dorsal ramus and a ventral ramus. The *dorsal ramus* contains nerves serving the epaxial region including skin, epaxial muscles and fasciae [11]. The dorsal ramus runs horizontally backwards, extends between the transverses processes and reaches the epaxial muscles. The *ventral ramus* leaves the paraspinal region to innervate the anterior part of the trunk and the limbs via the plexus, a network of interconnecting nerves.

**Fig. 5:**
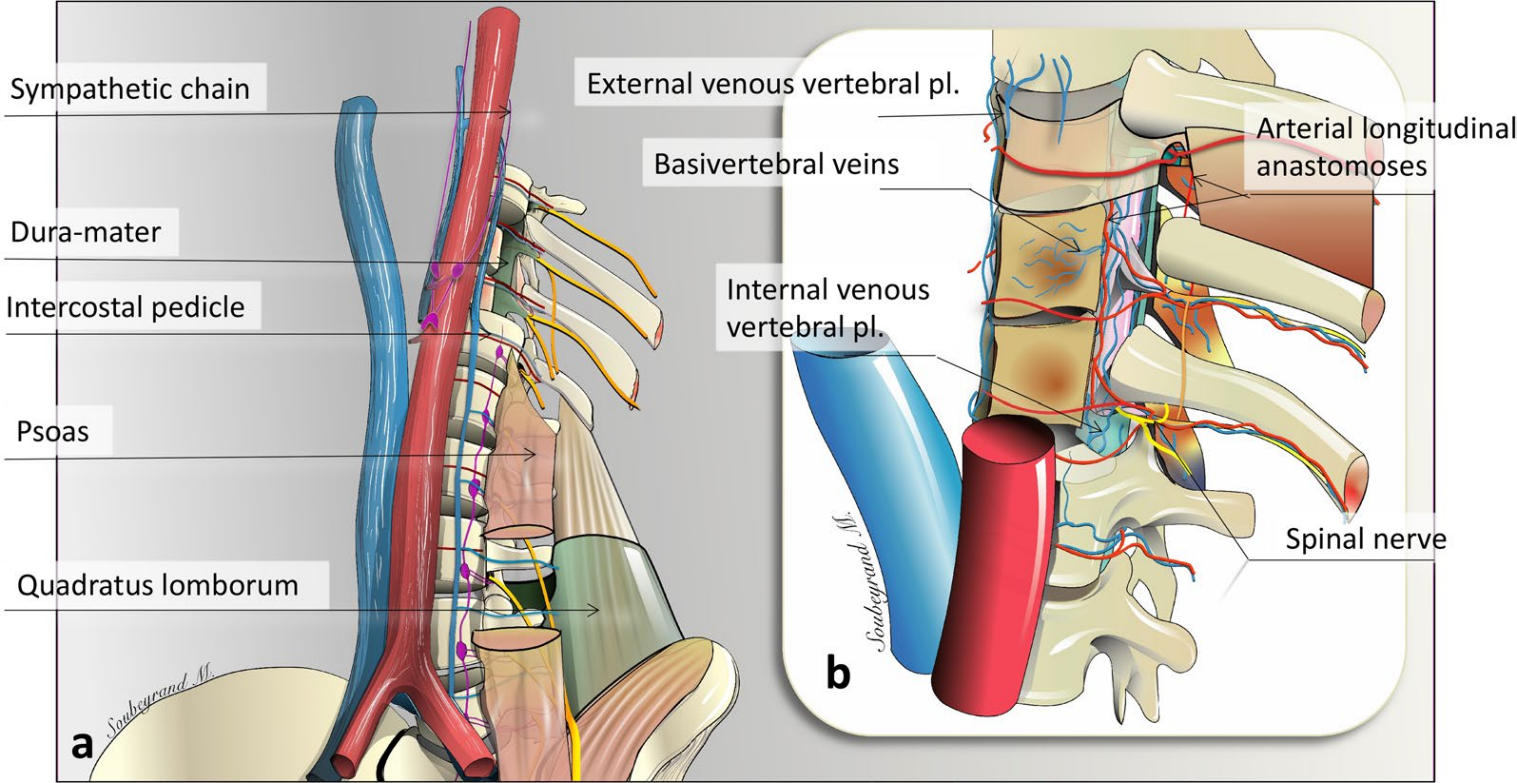
*Major nerves, veins and arteries of the paraspinal region*. **a** Anterior view of the thoracolumbar paraspinal region. **b** Enlarged view showing the Batson’s venous plexus and the origin and anastomosis of paraspinal arteries. Pl: plexus

The *sympathetic chain,* along which the *sympathetic paravertebral ganglia* are scattered*,* lies in close vicinity to the anterolateral aspect of the spine. Sympathetic ganglia are connected to the ventral ramus of the spinal nerve shortly after its emergence from the vertebral foramen via one or more *rami communicantes* (Fig. 5).

Perineural spread constitutes a pathway for paraspinal tumour extension [12]. The complex anatomy of the nerves explains the technical surgical difficulty of complete excision without spinal nerve or sympathetic chain injury. It is therefore extremely important that radiologists describe potential extensions into these neural structures since this impacts the functional prognosis before and after treatment.

### Arteries and veins of the paraspinal region

The *parietal arteries* and *veins* are segmentally arranged, bilaterally positioned pairs arising from the posterior aspect of the great vessels at each vertebral level. They supply paraspinal muscles, fasciae, ligaments, vertebrae and intervertebral discs (Fig. 5). This is the classical description, but these vessels can vary from this description [13].

At the thoracic and lumbar levels, the aorta gives off paired parietal arteries named *intercostal* and *lumbar arteries* [13]. They run laterally and posteriorly on the vertebral bodies underneath the sympathetic trunk to the intervals between adjacent transverse processes. Along their course, parietal arteries feed vertebral bodies through many branches. After the transverse processes, the parietal arteries give off a *dorsal spinal branch*. The dorsal spinal branch rapidly divides in two [14]. The prominent vessel supplies the cutaneous tissues*;* the remaining vessel forms a medial dorsal muscular anastomosis with the closest upper lumbar artery*,* which gives rise to a radiculomedullary artery.

Three* longitudinal anastomoses* (the anterior, ventrolateral and pretransverse anastomoses) located on the vertebral body and a long longitudinal dorsal anastomosis located posteriorly on the midline bridge two or three consecutive segmental arteries outside of the spine. They constitute a very efficient source of collateral circulation.

**Fig. 6:**
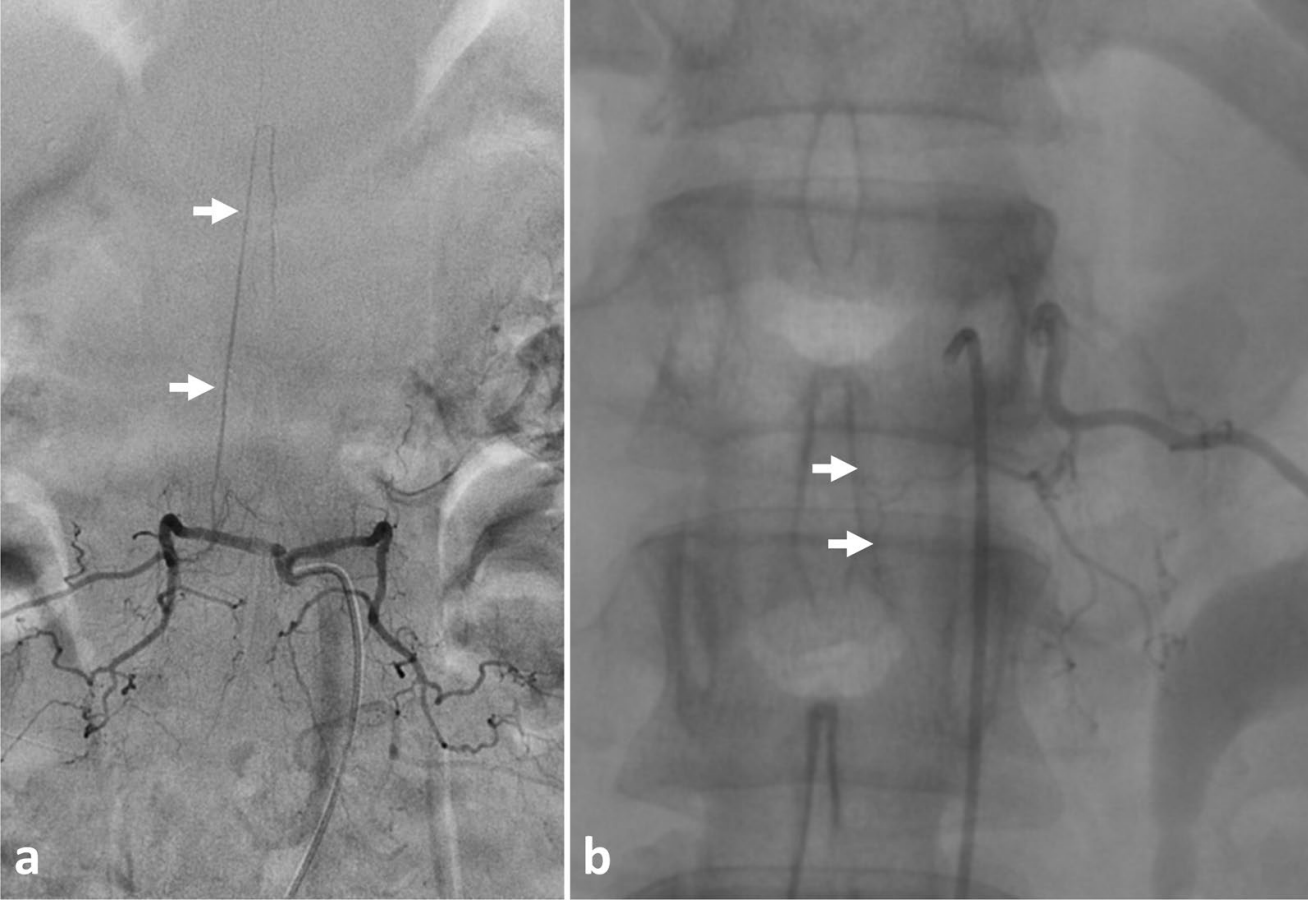
*Angiography of intercostal and lumbar arteries* performed before surgical excision of a neurofibroma at left T8-T9 level (**a**, **b**). The great radicular artery (Adamkiewicz) was originated from the distal part of the right 2nd lumbar arteries (**a**). Selective left intercostal angiogram shows a radiculomedullary artery originating from the left 5th intercostal artery (**b**)

**Fig. 7:**
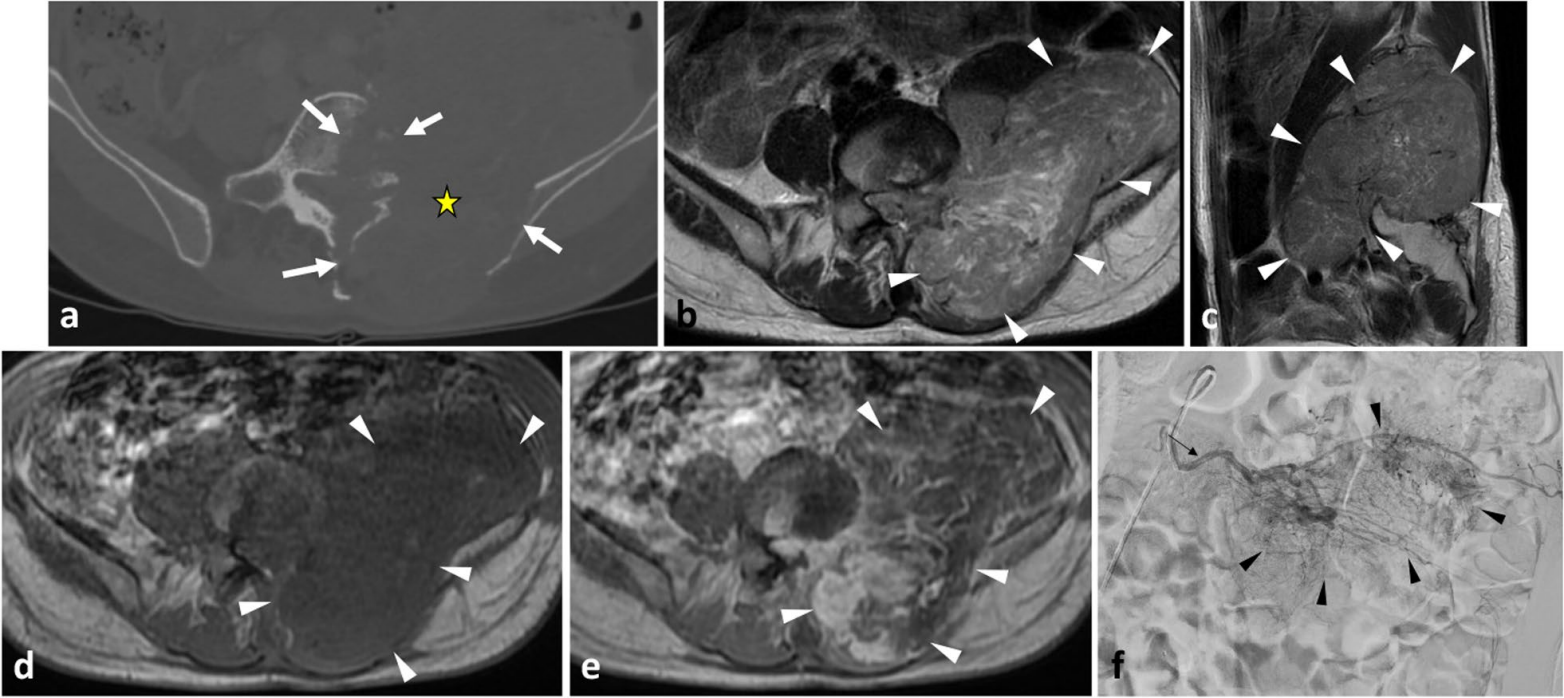
*Leiomyosarcoma* of left paraspinal muscles in a 65-year-old male with back pain and genitourinary issues. Contrast-enhanced axial CT scan reconstruction (**a**) demonstrates aggressive features with moth-eaten lysis of the vertebra and iliac bone (white arrows). The mass extends transversally in both epaxial and in hypaxial muscles (**b**–**e**, white arrowheads), and longitudinally along L2 to S1 (**c**). The mass has heterogeneous high signal intensity on axial (**b**) and sagittal (**c**) T2WI, low intensity signal on axial T1WI, and heterogeneous enhancement on contrast-enhanced fat saturation T1WI (white arrowheads) (**e**). Digital subtraction angiogram (**f**) of the left 3nd lumbar artery (arrow) shows hypervascularisation of the tumour (white arrowheads). The yellow star shows the epicentre of the mass

Certain paraspinal and spinal tumours are highly vascularised. Pre-embolisation angiograms can establish the vascular anatomy of the tumour (Figs. 6, 7), which allows careful selective cannulation of the arteries supplying the tumour. Angiography of segmental arteries is commonly used to localise the great anterior radiculomedullary artery (Adamkiewicz’s artery) prior to an *en bloc* spondylectomy. Knowing that roots ligation completely interrupts spinal cord blood supply at this level, it is believed that interruption of Adamkiewicz’s artery could cause a postoperative neurological deficit (Fig. 6) [15, 16].

The venous network of the spine is extremely dense and forms *three interconnecting plexuses* around the spine known as the Batson plexus: the *external venous vertebral plexus*, *internal venous vertebral plexus* and *basivertebral veins* (Fig. 5) [14, 17]. The *external vertebral venous system* surrounds the vertebral column and runs longitudinally from the cranial vault to the sacrum. The external vertebral venous system receives blood from paraspinal muscles. Similar to the disposition of paraspinal arterial vasculature in the paraspinal region, the vertebral plexus drains into segmental veins, meaning it drains first into a dorsal spinal vein and then into an intercostal or lumbar vein according to the spinal level. The vertebral venous system is dependent on either the caval system or the azygos system. The vertebral venous plexus is valveless and has a retrograde and bidirectional flow that provides a large capacity flow reserve. This explains the possibility of bidirectional locoregional tumour extension along the cephalocaudal axis and explains why metastases predominantly spread to the lungs.

### The paravertebral space

**Fig. 8:**
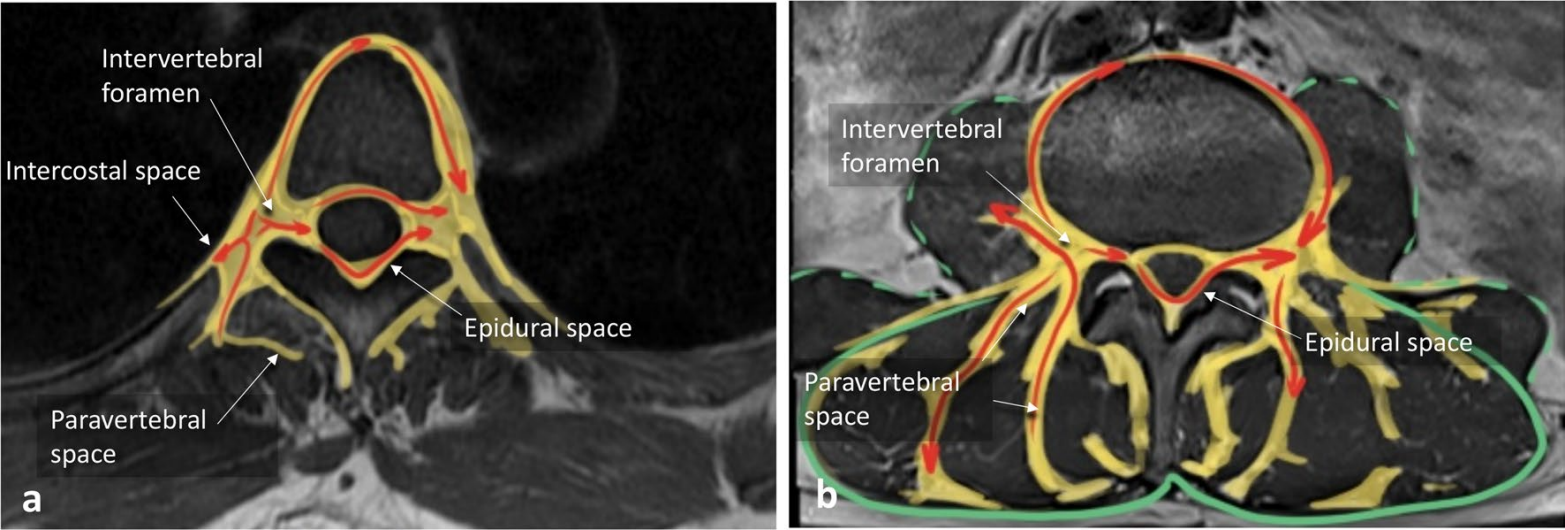
*Anatomy of the paraspinal space on axial T1WI.* Red arrows show the possible pathways involved in paraspinal tumours spreading

**Fig. 9:**
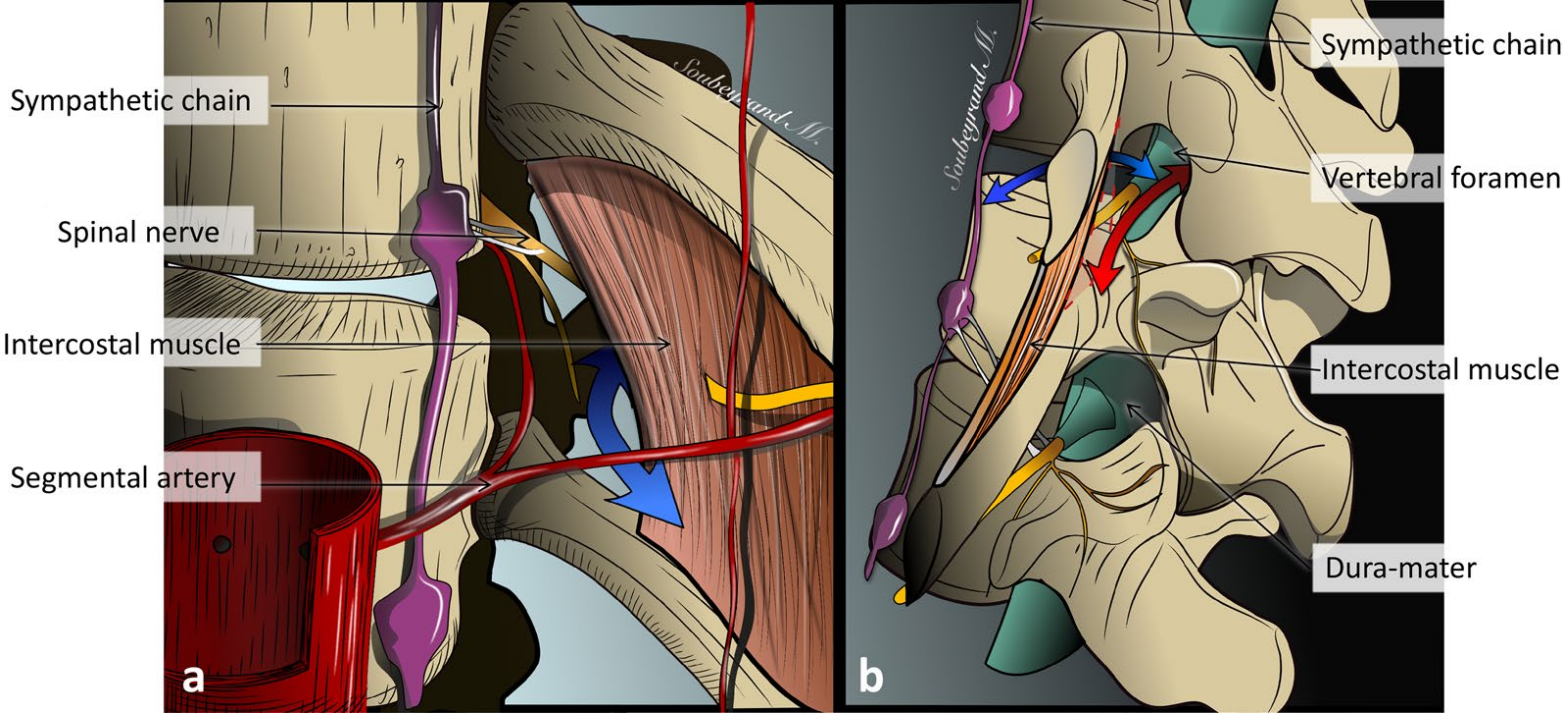
*Anatomy of the paraspinal space.* Anterior view of thoracic paraspinal region (**a**) Lateral view of the paraspinal region (**b**). The paraspinal region communicates with the intercostal space (blue arrow) and the epidural space (red arrow)

**Fig. 10:**
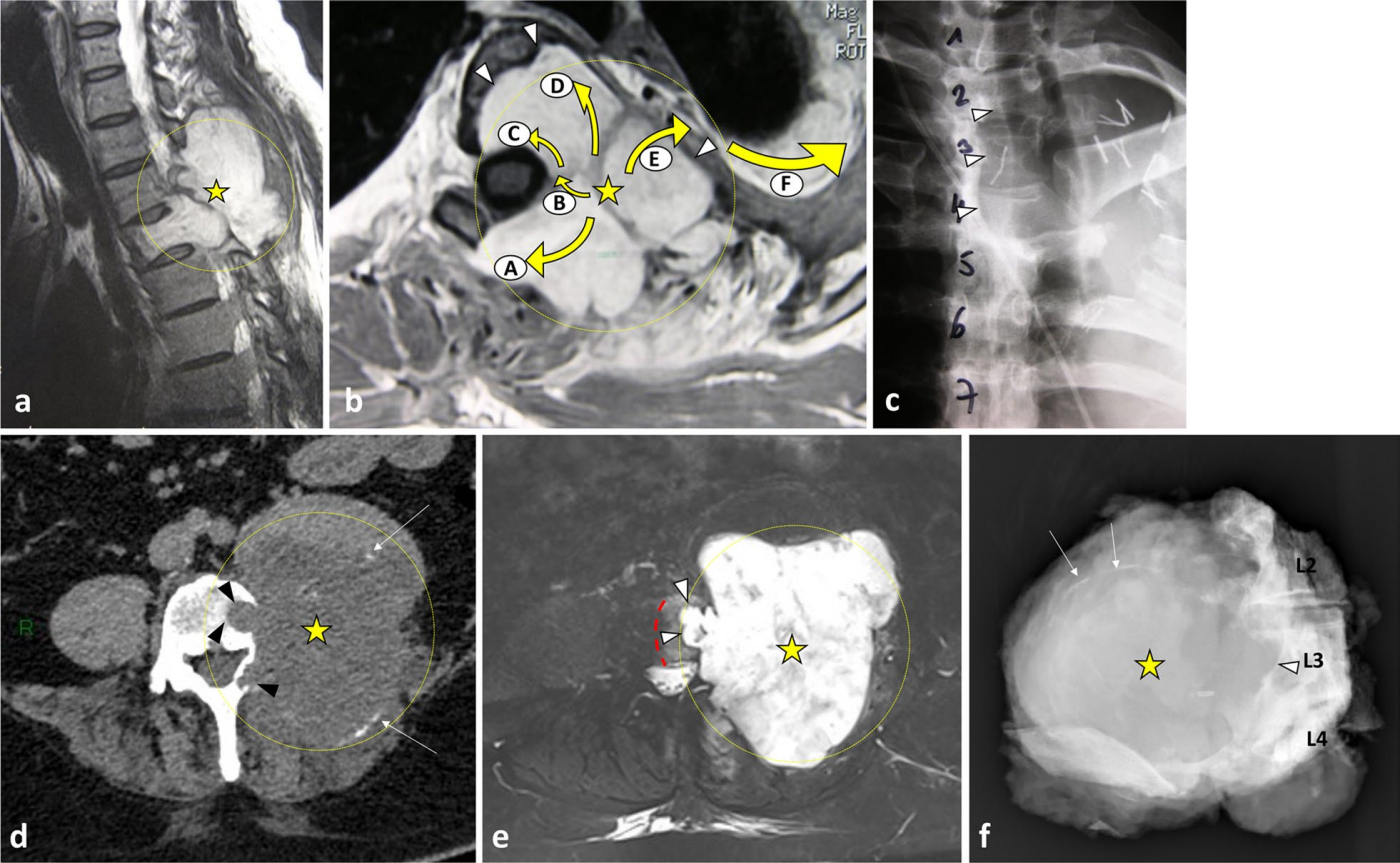
*Origin and extent of the paraspinal tumours. Myoepithelial soft tissue tumour* of paraspinal muscles in a 52-year-old male (with acute thoracic back pain) that secondary invades the 3rd thoracic vertebra and the 2nd, the 3rd and the 4th ribs (**a**, **b**, **c**). Sagittal T2WI (**a**) and axial T1WI with contrast agent (**b**) show a paraspinal tumour with an epicentre in epaxial muscles (star). Arrows show tumour’s spread towards the opposite side (**A**), into the bone (**D**), into the epidural space (**C**) via the intervertebral foramen (**B**), in the pleura (**F**) via the intercostal space (**F**). Postoperative anteroposterior radiograph shows a lysis of the 3rd and 4th vertebras (white arrowheads). The ribs were removed. *Chondrosarcoma* of the pedicle-transverse part of L3, that secondary invades the hypaxial and epaxial muscles, in a 62-year-old male (**d**, **e**). Axial CT reconstruction (**d**) and axial T2WI (**e**) show a mass with a cartilage matrix including calcifications (white arrows, (**d**)) and high T2WI intensity (**e**). The mass presents aggressive bone lysis (arrowheads) surrounded by inflammation (red dashed line). Posteroanterior radiograph of the surgical sample of vertebrectomy and pelvic resection (**f**) shows a shadow and calcification (arrows) and lysis of the vertebral body and transverse process of L3 (arrowhead)

**Fig. 11:**
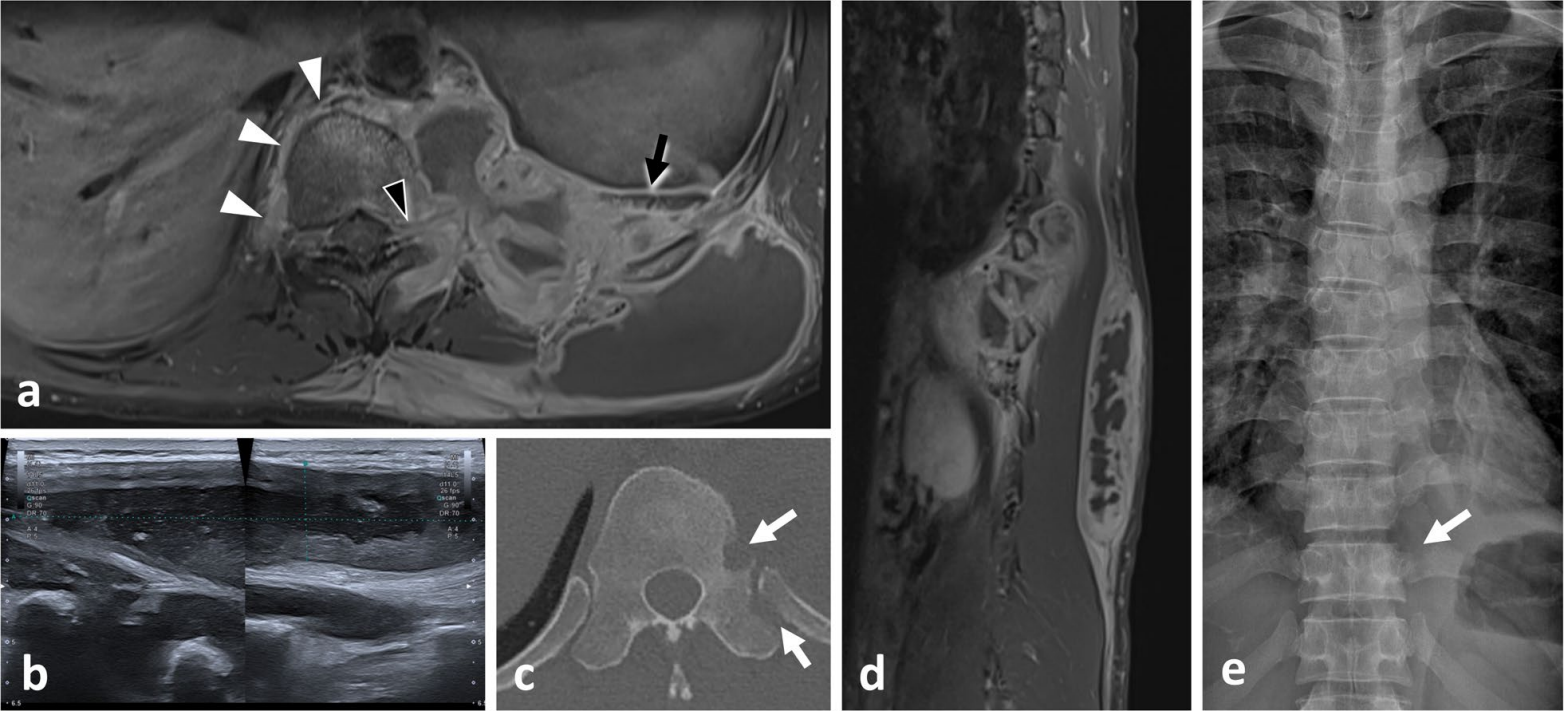
*Cold abscesses* in a 37-year-old male with mild thoracic pain, weight loss and fatigue for 1 month. Axial (**a**) and sagittal (**d**) T1WI with contrast agent show large left paraspinal abscesses, which extend in both hypaxial and epaxial (superficial and deep) regions. Abscesses extend along the vertebral body to the contralateral side (white arrowheads) and into the left vertebral foramen (black arrowhead). Axial CT scan reconstruction (**c**) and anteroposterior radiograph (**e**) show septic arthritis of the costovertebral joint and spondylitis (white arrows). Anteroposterior radiograph shows the left hypaxial mass along the left side of the side (black arrows). As the lesion was superficial, US was used for percutaneous guided biopsy (**b**). US shows a hypoechoic mass with posterior acoustic enhancement

The paravertebral space is of major importance because it constitutes a corridor for paraspinal lesion extension. It represents the adipose compartment distributed between the paraspinal muscles and along paraspinal vessels and nerves (Fig. 8). At the intervertebral foramen, the paraspinal space opens into the extradural neural axis compartment or epidural space [12]. At the thoracic level, the paraspinal space is contiguous with the intercostal spaces, allowing tumour extension (Figs. 9, 10). Contralateral spread can occur anteriorly to the vertebral body as well as towards the extradural neural axis (Fig. 11). Lastly, cervical and thoracic, as well as thoracic and lumbar paravertebral spaces, are anatomically connected, facilitating cephalocaudal extension.

## Clinical features

Superficial masses might present as a bump in the back. Clinical symptoms of deep paraspinal masses are non-specific and their onset can be acute, subacute or chronic [18]. This lack of specificity can delay diagnosis. Symptoms occur late in patients, when the tumour reaches a considerable size and invades critical structures including bone or nerve/spinal cord by extension into the intervertebral/vertebral foramen. Symptoms include axial back pain, usually associated with some indicators of serious spinal pathology (red flags), including bladder or bowel dysfunction, history of neoplasia, presence of other medical conditions, fever, thoracic location and nocturnal pain. Range of motion limitations, torticollis or scoliosis can be observed. Myelopathic symptoms and radicular pain are, respectively, related to intraspinal extension and foraminal extension of the tumour, respectively. Rarely, paraspinal lesions are revealed by vertebral fracture or, in the case of an hypaxial lesion, by abdominal pain. Tables 4 and 5 report specific clinical features associated with certain conditions.

## Imaging modalities

Imaging provides critical information and gives the multidisciplinary team (MDT) a comprehensive description of the lesion on which to base their indications. Multimodality imaging is essential to characterise both paraspinal soft tissue involvement and skeletal changes and should always be performed before a biopsy [19, 20].

### Radiographs

**Fig. 12:**
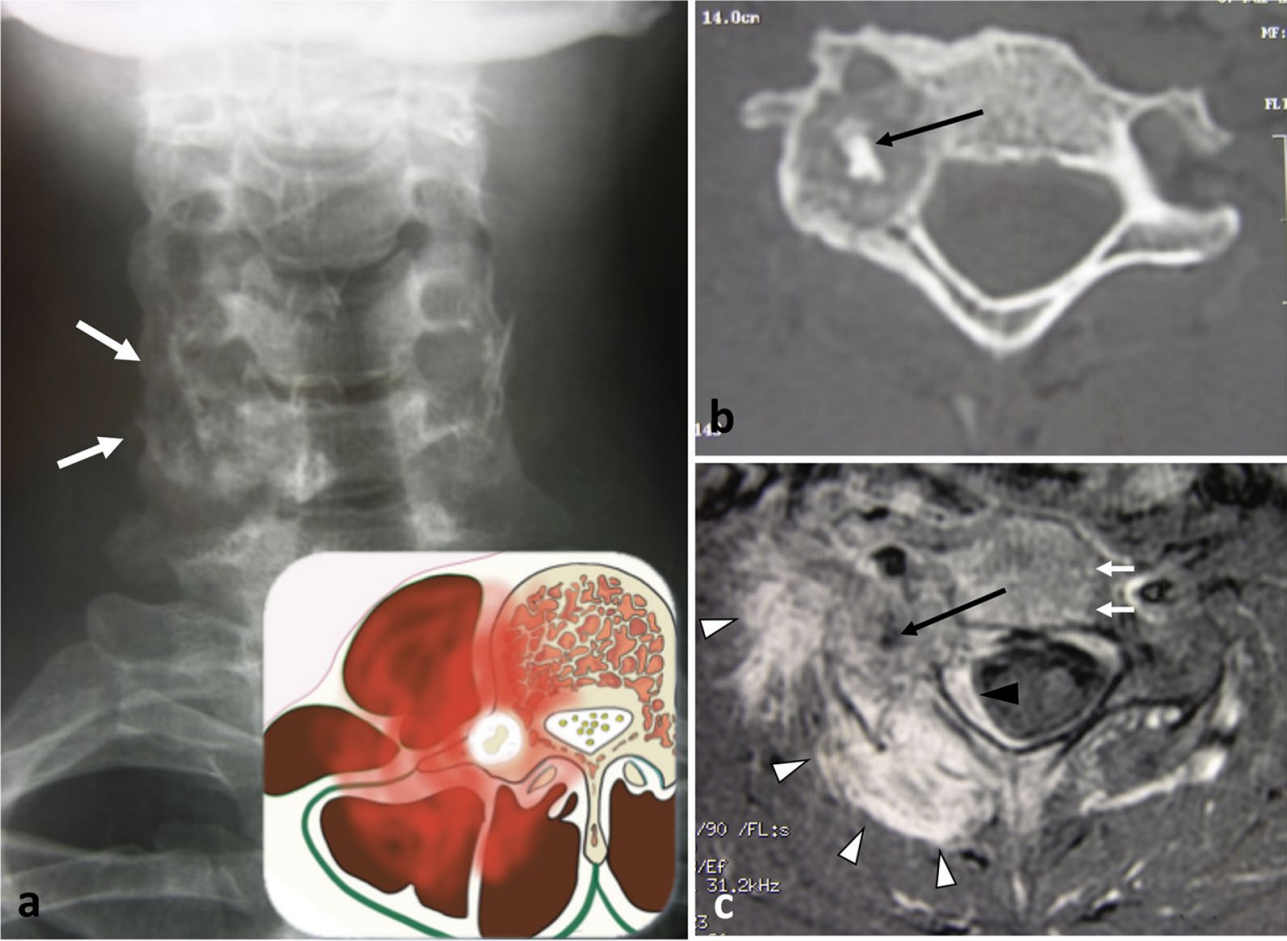
*Spinal osteoblastoma* of the C6 in a 36-year-old male with intense cervical pain, worse at night, and decreased range of motion. Anteroposterior radiograph of the cervical spine (**a**) shows a mixed lesion of the right transverse process of C6 (white arrows). Axial CT reconstruction (**b**) scan shows a lytic lesion with an internal calcification (black arrow) and a rim of sclerosis (black arrowhead). Gadolinium-enhanced fat-suppressed axial T1WI (**c**) shows heterogeneous enhancement of the mass with associated enhancement of the surrounding paraspinal soft tissues termed ‘the flare phenomenon’ (white arrowheads). The mass invades the epidural space (black arrowhead)

**Fig. 13:**
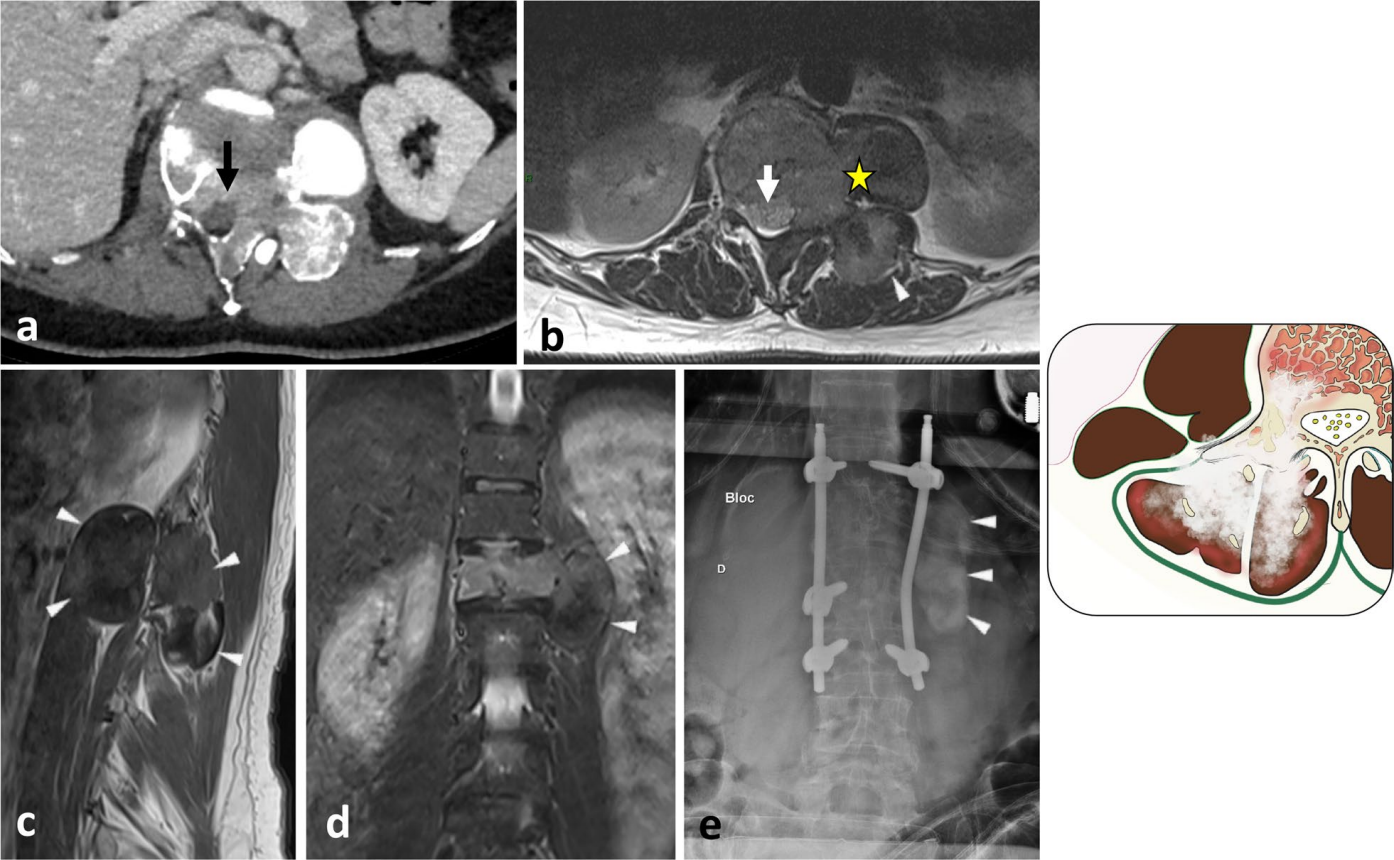
*Spinal osteosarcoma* in a 23-year-old male with back pain and progressive weakness of both lower limbs. Contrast-enhanced axial CT scan reconstruction (**a**) shows corporeal-pedicle bone formation of L1. The presence of a fat plane between the tumour and the epaxial muscles (arrowhead) suggests that the mass turn away the epaxial muscles rather that invades them. The mass has non-specific imaging features on MRI: mild hyperintense on axial T2WI (**b**), hyposignal on sagittal T1WI (**c**), mild and heterogeneous enhancement on contrast-enhanced, fat-suppressed T1WI (**d**). The mass also invades epidural space (arrows). Anteroposterior radiography (**e**) performed after pedicle screw fixation showing bone formation in the left paraspinal region. Yellow star shows the epicentre of the mass

Although the value of radiographs in evaluating paraspinal lesions is limited—most of the relevant information being better explored with computed tomography (CT)—they should not be ignored (Figs. 11, 12, 13) [18, 21]. Firstly, a standing radiograph is the only modality that gives a functional overview of the spine and provides insight into the overall morphology of the spine. Secondly, it shows whether the lesion is visible on plain radiographs, and indeed on intraoperative radiographs which may be used to guide surgical procedures. Rarely, dorsal and lumbar spine masses can be seen as paravertebral soft tissue shadows (Fig. 11).

### Ultrasound

**Fig. 14:**
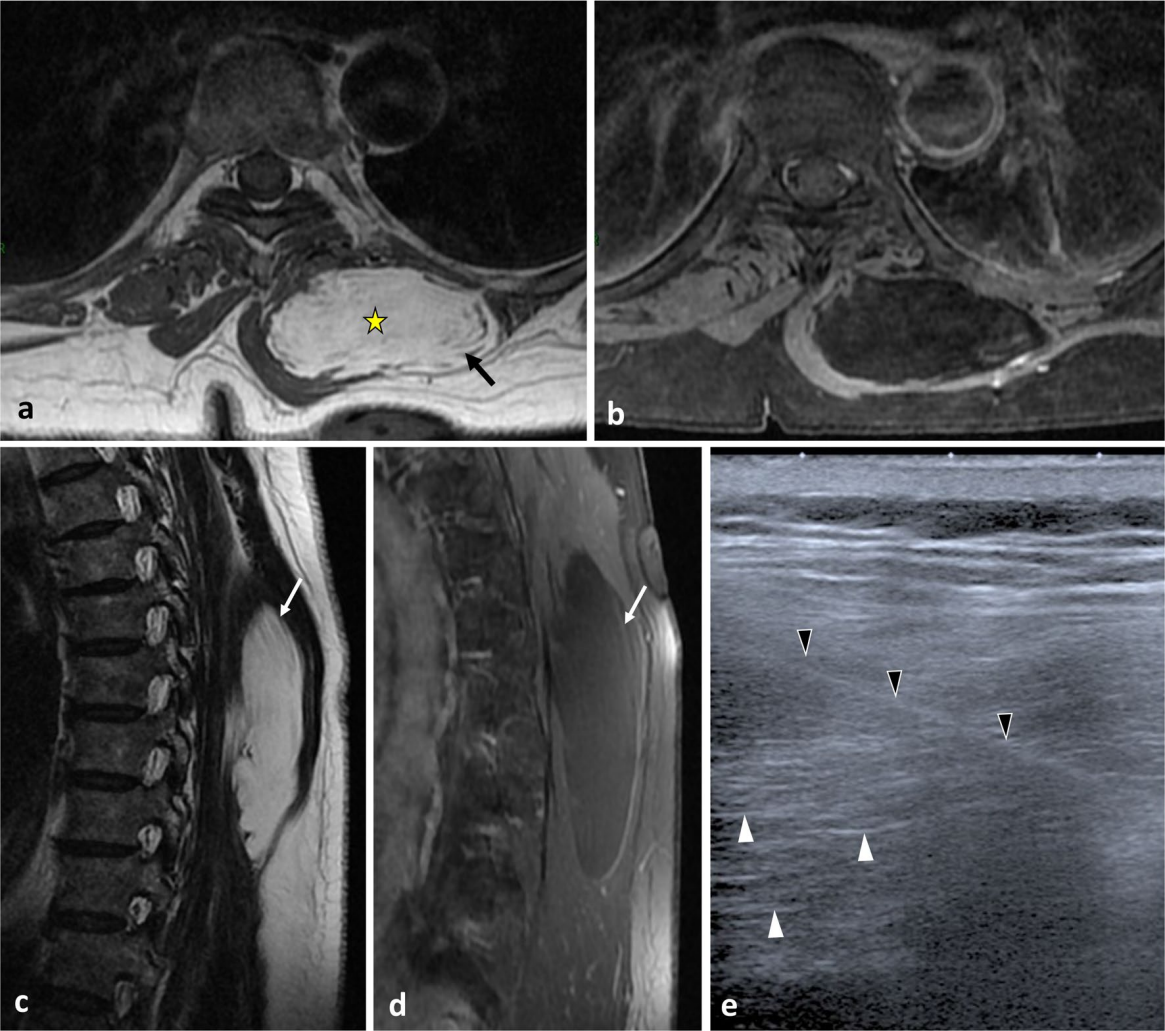
*Intramuscular lipoma* in the left erector spinae muscle in a 60-year-old male with soft, painless and non-inflammatory paraspinal tumefaction. MRI demonstrates the typical features of intramuscular lipoma: high intensity on T1WI (**a**), saturation on fat-saturated T1WI (**b**), high intensity onT2WI (**c**) and absence of enhancement on contrast-enhanced fat saturation T1WI (**d**). Streaky structures correspond to entrapped muscle fibres (arrows). US-guided biopsy (**e**, black arrowheads) shows a hyperechoic mass with thin transversal lines (white arrowheads). Pathological report demonstrates negative detection for MDM2 supporting classification as lipoma instead of atypical lipomatous tumour. The yellow star shows the epicentre of the mass

**Fig. 15:**
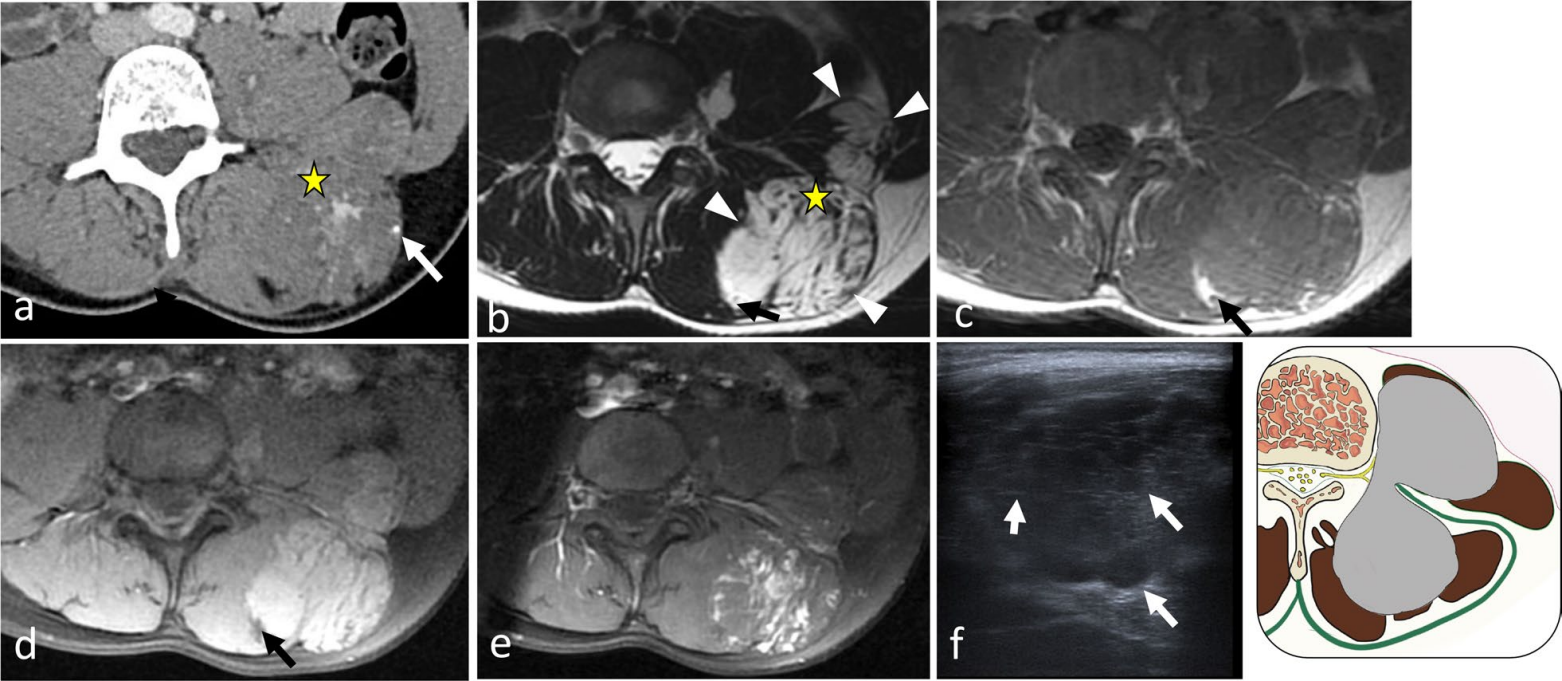
*Haemangioma* in a 37-year-old female presenting with mild paraspinal pain and soft and slow-growing tumefaction. Contrast-enhanced axial CT scan reconstruction (**a**), axial T2WI (**b**) and axial T1WI (**d**) show a bilobed mass in the erector spinae muscles, the psoas muscle and the quadratus lumborum muscle with phlebolite (white arrow) and fat content (black arrow). Contrast-enhanced axial T1WI with fat saturation (**e**) demonstrates heterogeneous enhancement (**a**). On US, the mass appears as an ill-defined hypoechoic mass with multiple spaces within (white arrows) (**f**). The yellow star shows the epicentre of the mass

**Fig. 16:**
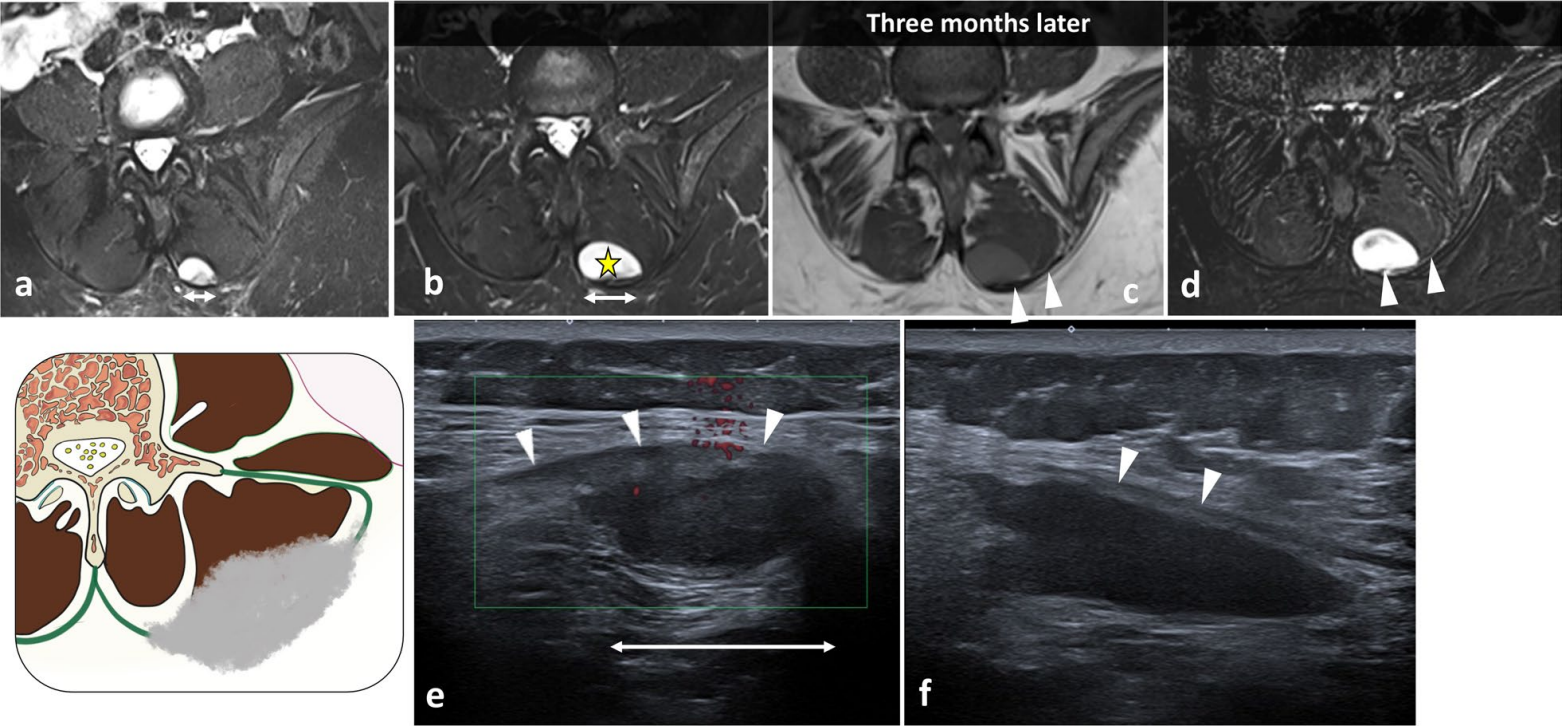
*Desmoid tumour* that extends along the left erector spinae aponeurosis in a 39-year-old male with recent trauma and pain. T2WI demonstrates a circumscribed mass with high intensity (**a**). MRI performed 3 months later shows a fast-growing tumour (**b**, **c**, **d**). The desmoid tumour is hyperintense to muscle in T2WI (**b**) and T1WI (**c**) and enhances on contrast-enhanced T1WI (**d**). US performed in transversal (**e**) and longitudinal plane (**f**) shows a well-defined hypoechoic mass onto the erector spinae aponeurosis (arrowheads). The yellow star shows the epicentre of the mass

Ultrasound (US) is particularly valuable in the initial screening of superficial and palpable paraspinal tumours [22]. US allows cystic masses to be distinguished from solid masses and can determine the morphological features of the mass (size, shape, number, margin) and anatomical relationships with fasciae and skin (Figs. 11, 14, 15, 16). Some tumours may have specific US features like fibrous and lipomatous tumours. In addition to B-mode, the analysis of the biomechanical properties and of the vascularity of the lesion can be used to refine its characterisation [23]. Elastography had good performance in detecting or excluding malignant soft tissue tumour with a sensitivity estimate between 0.72 and 0.83 and a specificity of 0.60 and 0.82 for shear wave- and strain elastography, respectively [24]. Unsmooth margin and high vascular density are independent predictors for malignancy [25, 26].

### CT imaging

**Fig. 17:**
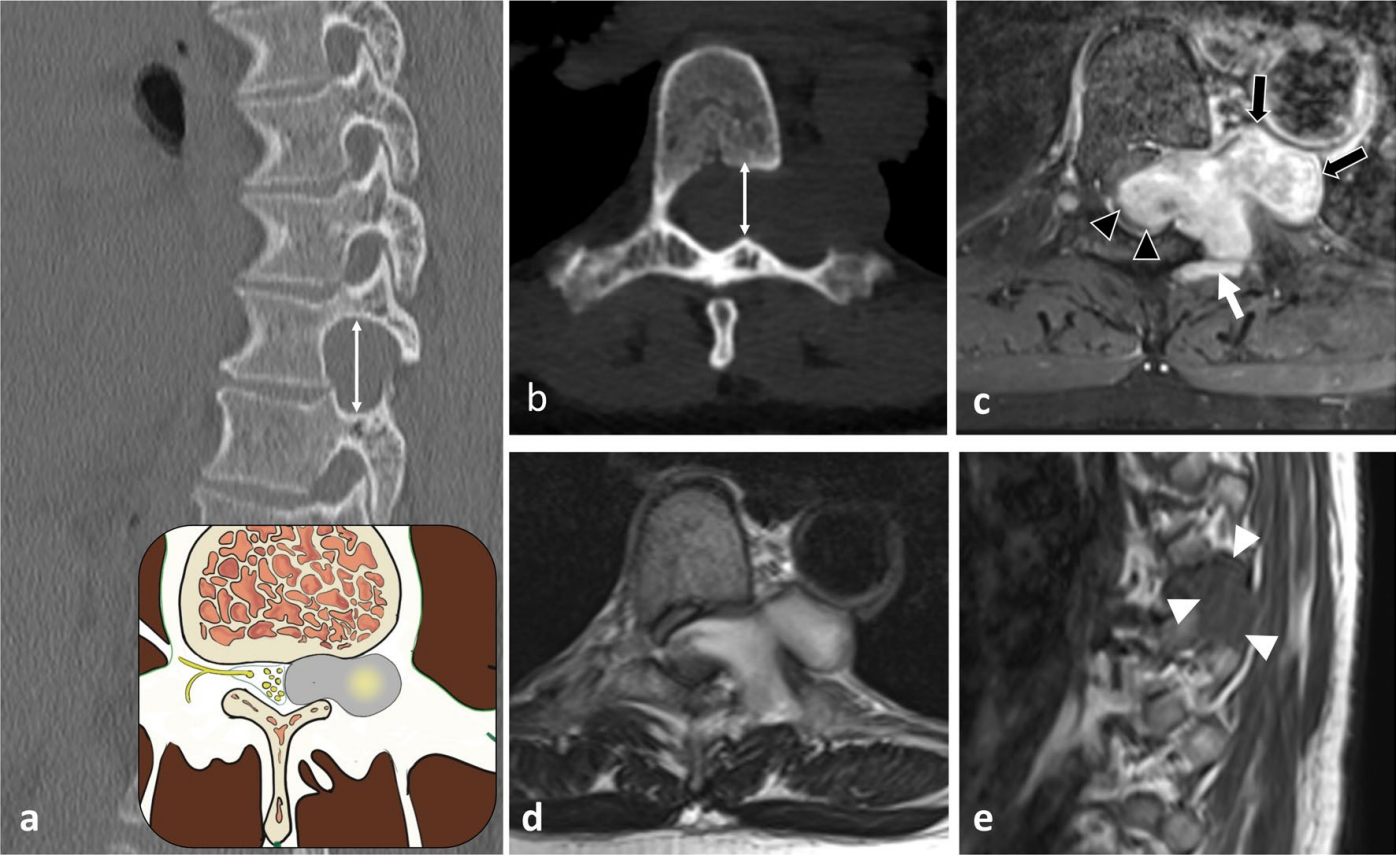
*Neurofibroma* of the 7th left spinal nerve in a 36-year-old female with thoracic radicular pain. Sagittal (**a**) and axial CT reconstruction (**b**) shows the widening of neural foramen (double arrows). Axial CT (**b**), axial T1WI with contrast (**c**) and axial T2WI (**d**) demonstrate the typical dumbbell configuration of nervous tumour with intraspinal (back arrowheads) and paraspinal components (black arrows) that communicate via the intravertebral foramen. The mass presents high signal intensity on T2WI (**d**), low signal intensity on T1WI (**e**) and homogeneous enhancement (**d**). The nervous mass extends into the multifidus muscle (white arrow) and the intercostal space (white arrowheads). The yellow star shows the epicentre of the mass

**Fig. 18:**
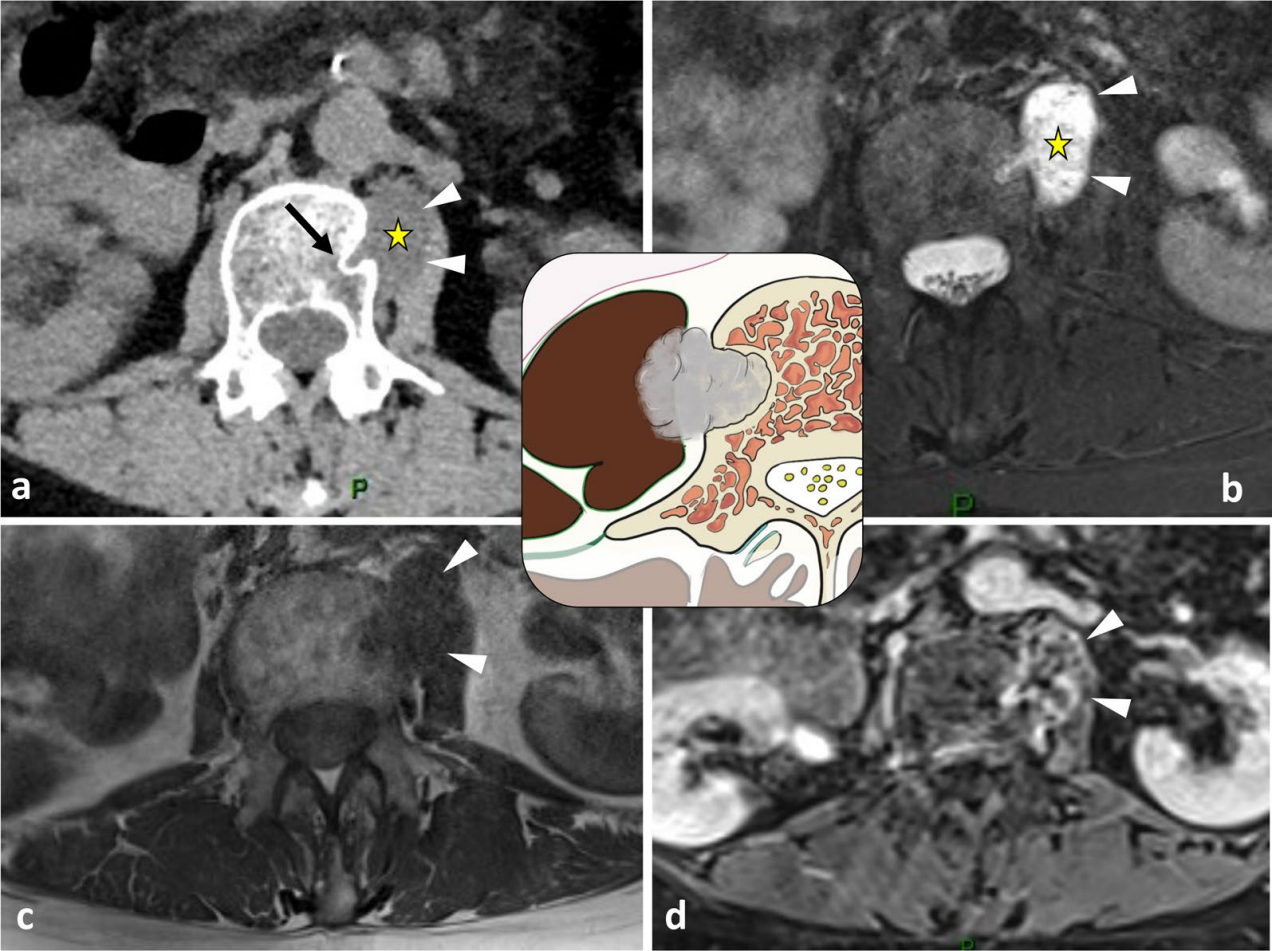
*Schwannoma* of a ventral branch of the right 2nd lumbar spinal nerve in a 32-year-old female with neuropathic pain of the right anterior medial thigh. Axial CT reconstruction (**a**) shows a hypodense mass within the psoas muscle (white arrowhead) with high signal on T2WI (**b**), low signal on T1WI (**c**) and heterogeneous contrast-enhanced fat saturation T1WI (**d**). The mass extends into the vertebral body forming a lytic lesion with sclerotic margin (black arrow). The yellow star shows the epicentre of the mass

CT is useful in evaluating the paraspinal mass matrix and adjacent bone changes (Figs. 12, 13, 15). CT evaluates the presence of soft tissue calcifications or ossifications within the tumour and determines its morphological characteristics (osteoid, chondroid, dystrophic) [21]. Bone changes, including exostosis formation, periosteal reactions, extrinsic cortical erosion (scalloping) or foramen enlargement, reflect the biological activity of the lesion. Low-level biological activity is associated with slow-growing lytic lesions or bone scalloping with sclerotic margins or widening of the neural foramen (Figs. 17, 18). Aggressive lesions cause ill-defined bone destruction, erosion or periosteal reactions. In cases of lytic destruction of the vertebrae, CT can assess fracture risk and instability.

### Magnetic resonance imaging

Magnetic resonance imaging (MRI) is the gold standard for accurate assessment of the size and extent of paraspinal lesions (Figs. 4, 10). It provides better soft tissue contrast resolution than other imaging modalities [19]. MRI is also useful for evaluating bone changes, particularly bone marrow replacement and cortical or periosteal extensions. MRI allows tumour staging and detection of neurovascular involvement that informs preoperative planning.

MRI must be performed in two orthogonal planes and includes T1-weighted and T2-weighted sequences with fat suppression [27]. The axial plane is essential to determine the compartmental anatomy and whether the tumour is invading or encasing surrounding structures. A sagittal plane is required to specify the longitudinal extension and anatomical landmark of masses. The slices have to extend sufficiently laterally to cover all the paraspinal soft tissues. The coronal plane could also be performed when the lateral extension of the disease is very important.

T1-weighted sequences are valuable for determining the anatomy of the mass. The intrinsic signal characteristics on T1-weighted images (T1WI) and T2-weighted images (T2WI) are often non-specific. However, in some cases, signal intensity characteristics, which are related to the histological features of the tumour, might be effective for tissue characterisation, in particular to provide an insight into the lipomatous, fibrous or cellular nature of the mass. For instance, hypercellular or myxoid lesions generally follow fluid signal intensity, whereas collagenous or fibrous lesions usually have low/intermediate signal intensity on T2WI. Masses containing fat, methaemoglobin, proteinaceous fluid or melanin have high signal intensity on T1WI. Post-contrast T1 images are helpful to distinguish cystic from solid tissues, to demonstrate the vascularity of the mass and to identify tumour necrosis, the latter two criteria being suggestive of malignancy (Figs. 4, 7).

Other sequences should be carried out on a case-by-case basis and can help finalise the characterisation of a lesion.

A T2-weighted gradient-echo sequence might be considered to assess for the presence of haemosiderin.

Diffusion-weighted imaging allows a tumour’s cellularity to be assessed. Malignancy is usually suspected if high cellularity, i.e. low diffusion, is detected. However, the distinction between benign and malignant soft tissue tumours is hindered because some apparent diffusion coefficient values for benign and malignant soft tissue tumours can overlap [28].

By characterising tissue perfusion, capillary permeability and interstitial space volume, dynamic contrast-enhanced MRI may help differentiate between benign (low-slope) and malignant (high-slope) tumours. However, its diagnostic contribution remains limited because some slope values for benign and malignant lesions overlap [29].

## Practical approaches to analysis of paraspinal tumour images

Paraspinal tumours can develop either from the spine or from neighbouring soft tissues (Fig. 10). The therapeutic strategy is determined after analysing the tumour’s origin and extent.

Before imaging analysis, *anamnesis* is a key element of the diagnostic investigation. For many paraspinal tumours, clinical data such as age and history can be highly suggestive of their nature (Table 1).

**Table 1:** Differential diagnosis of paraspinal masses by medical history

Patient history	Diagnostic assumption
History of primary tumour	Metastasis
History of chronic anaemic states (sickle cell disease, thalassemia)	Extramedullary haematopoiesis
History of neurofibromatosis	Nerve sheath tumour
History of trauma	Haematoma Fat necrosis Fibromatosis Myositis ossificans
Endemic region Immunosuppression	Tuberculosis
Immunosuppression	Lymphoma

**Fig. 19:**
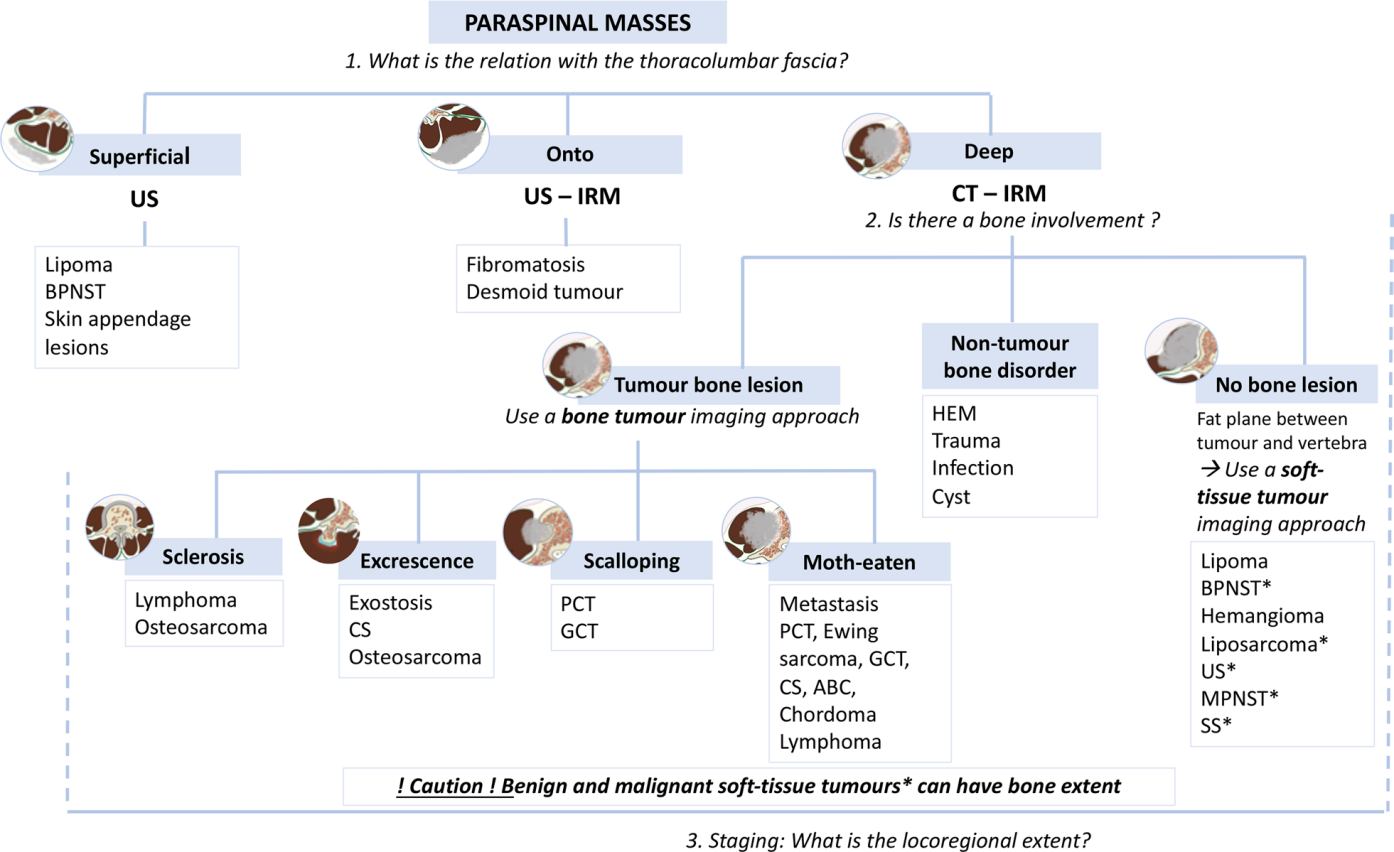
*Flowchart for paraspinal masses.* Stars represent soft tissues tumours with bone involvement. BPMNST, benign peripheral nerve sheath tumours; MPNST, malignant peripheral nerve sheath tumours; ABC, aneurysmal bone cyst; CS, chondrosarcoma; GCT, giant cell tumours; PCT, plasma cells tumours; US, undifferentiated sarcoma; and SS, synovial sarcoma

**Fig. 20:**
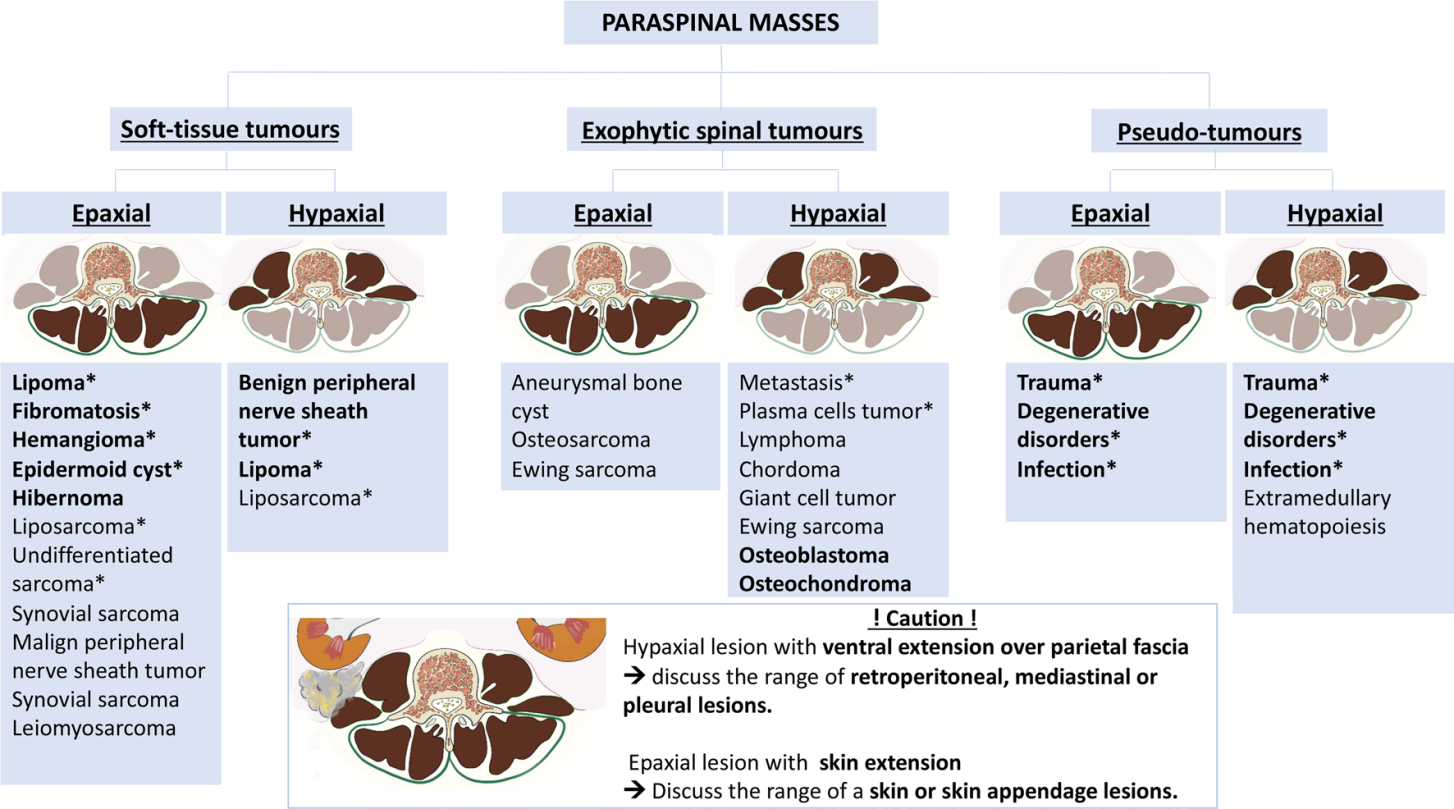
*Overview of the paraspinal masses*. Stars represent most frequents lesions. Words in bold represent benign lesions. BPMNST, benign peripheral nerve sheath tumours; MPNST, malignant peripheral nerve sheath tumours; ABC, aneurysmal bone cyst; CS, chondrosarcoma; GCT, giant cell tumours; and PCT, plasma cells tumours

The first step of imaging analysis is to *localise the epicentre* of the mass: hypaxial or epaxial. For epaxial lesions, its location relative to the TLF allows superficial tumours to be distinguished from deep tumours (Fig. 19). In our experience, tumours of neural origins and liposarcomas are the most frequent tumours in the hypaxial region (Fig. 20). Epaxial tumours are dominated by lipomas and fibrous tumours. In the particular case of spinal and paraspinal lesions, the epicentre rule is not absolute, especially for expansile vertebral tumours with paraspinal extension. Vertebrae are irregular short bones with low bone mineral density and thin cortical thickness, especially in vertebral bodies [30]. Bone tumours can therefore effortlessly cross the cortex and grow outside the bone. Subsequent growth of tumours in the soft tissues may shift the epicentre of the mass laterally within the paraspinal region. A hypaxial lesion that extends far in front to the parietal fascia should be considered the range of retroperitoneal, pleural or mediastinal lesions according to the vertebral level. The range of skin lesions or skin appendage lesions should be considered when an epaxial lesion extends to the skin especially as the upper back is a common site for skin appendage lesions [31].

Hence, the second step is to determine whether the paraspinal tumour originates from soft tissue elements rather than from bone via* analysis of vertebral bone changes* [32, 33]. Soft tissue tumours involving the spine generally do not extend into vertebrae; instead, expansible spinal tumours, either benign or malignant, have locally aggressive imaging features and invade the paraspinal region. Also, the analysis of the overall bone structure and vertebral joints would provide preliminary insight into a metastatic process or tumour mimic including infection, arthritis or extramedullary haematopoiesis. Vertebral scalloping and widening of the neural foramen suggest a peripheral nerve sheath tumour (PNST).

The next step is to *assess the aggressiveness* of the mass and potentially *its histological nature*. The aggressiveness of paraspinal tumours is characterised by the same radiological criteria as any other soft tissue masses [19, 34]. Thus, features evocating a malignant soft tissue tumour include large diameter (> 5 cm), deep location, heterogeneous signal/density with necrosis, and haemorrhage. Aggressive periosteal reactions and permeative/patchy bone destruction are warning signs of aggressiveness for both spinal and soft tissue tumours.

Finally, radiologists must *define the locoregional and distant extension* of masses. This is essential for determining tumour management. The longitudinal extent of paraspinal tumours is recorded in terms of the segments of vertebrae involved. Note that spinal tumours have a greater tendency to spread transversely, while tumours arising from the paraspinal soft tissue are more likely to spread longitudinally. Locoregional extension of soft tissue masses is defined based on location, size, volume of peritumoural oedema, neurovascular involvement, extension into the adjacent bone and extension into other compartments or organs.

Neurovascular status, and most importantly involvement of the spinal nerves, is determined by the disappearance of the fat plane, which has high intensity on T1WI, separating the neurovascular pedicle and the tumour. Spinal nerve invasion, especially in the lumbar region, informs surgical planning.

Bone involvement, whether from secondary invasion of a paraspinal soft tissue tumour or a bone tumour of the spine, determines the surgical planning. Radiologists must try to distinguish peritumoural inflammation from tumour infiltration. While osseous invasion by soft tissue tumours including sarcomas is classically considered to be uncommon in limbs, paraspinal soft tissue tumours, even benign, seem to more frequently impact the adjacent vertebra or rib (Table 2; Additional file 1: Fig. S1) [28, 35, 36]. We hypothesise that the thin periosteum of vertebrae offers less resistance to tumour invasion than the thick periosteum of long bone [35]. On CT imaging, benign tumours exhibit cortical scalloping with a sclerotic rim (Fig. 18); aggressive tumours display bone destruction with aggressive periosteal reactions (Fig. 7). On MRI, in case of paraspinal soft tissue tumours involving the spine, the disappearance of paravertebral fat adjacent to the vertebra on T1WI and bone enhancement on T1WI fat suppressed are warning signs for vertebral invasion, but they have low specificity. Reciprocally, in case of spinal tumours extending into the paraspinal compartment, the presence of paravertebral fat adjacent to the muscle indicates the absence of tumour invasion. Cortical abutment on T1WI and periosteal signals on T2WI are also features suggestive of periosteal infiltration by soft tissue tumours. Note that abnormal signal intensity in a vertebral body adjacent to a paraspinal tumour may be secondary to either direct invasion or reactive inflammatory changes. Hyposignal on both T2WI and T1WI allows to distinguish invasion from reactive inflammatory changes [37]. Spinal stability is a key component in the management of a bone lesion and must be assessed using the Spine Instability Neoplastic Score (SINS), which includes the following factors: global spinal location of the tumour, pain, bone lesion quality, spinal alignment, vertebral body collapse and posterior involvement [38].

**Table 2:** List of paraspinal soft tissue tumours with possible bone involvement

Condition	Description of bone change
*Soft tissue tumours*	
Benign	
BPNST	Geographic with sclerotic margin or indistinct borders, foramen enlargement
Leiomyoma	Geographic indistinct borders
Myxoma	Geographic indistinct borders
Locally aggressive	
Solitary fibrous tumour	Geographic indistinct borders
Malignant	
MPNST	Geographic indistinct borders or moth-eaten lysis
Liposarcoma	Moth-eaten lysis
Leiomyosarcoma	Moth-eaten lysis
Synovial sarcoma	Moth-eaten or geographic with sclerotic margin; periosteal reaction
*Tumours mimics*	
Trauma	Fracture, luxation
Infection	Erosion of vertebral body endplates and disc space narrowing
HEM	Diffuse changes: sclerotic with areas of lucency, vertebral deformity (fish mouth, H-shaped)
Crystal deposition disease	Erosion with sclerotic margin, other affected joints
Degenerative	Vertebral narrowing, approximation of adjacent spinous processes, facet joint hypertrophy, sclerosis, osteophytes

**Fig. 21:**
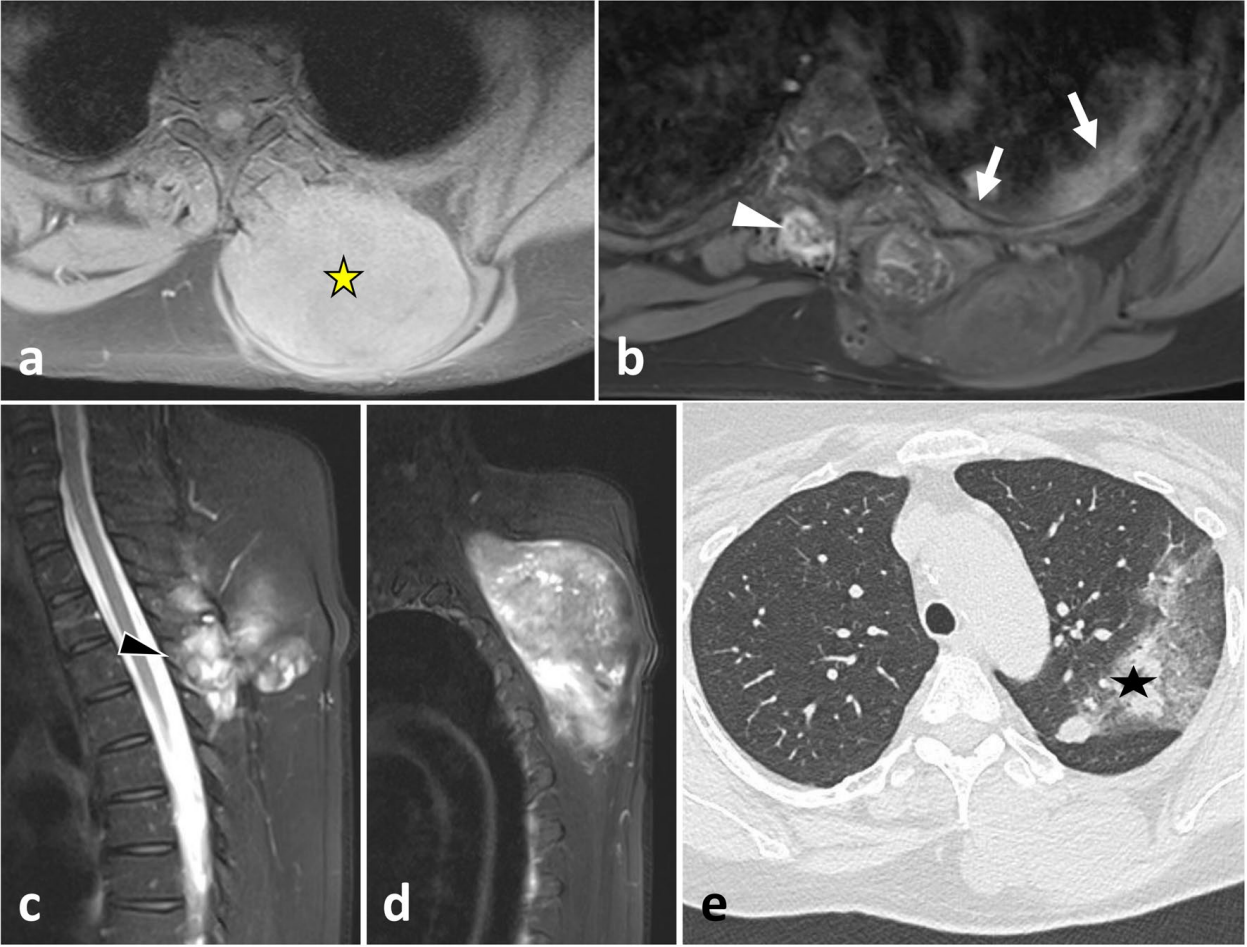
*Undifferentiated sarcoma* of left thoracic paraspinal soft tissues in a 42-year-old female with severe non-mechanical thoracic pain and palpable hard mass. Axial fat-suppressed T1WI (**a**) and gadolinium-enhanced T1WI (**b**) show a necrotic mass in bilateral hypaxial muscles. Sagittal STIR WI (**c**, **d**) shows paraspinal extension along five segmental levels. Sagittal T2WI shows bone inflammation of the spinous process (black arrowhead). Axial fat-suppressed T1WI and axial CT scan (**e**) demonstrate tumour’s spread towards the opposite side (white arrowhead), pleural extension (white arrows) and pulmonary extension (star). The yellow star shows the epicentre of the mass

As described earlier, due to poor anatomical boundaries, hypaxial tumours can expand into the mediastinum and retroperitoneum and invade the structures within, particularly the great vessels, thoracic duct and pleura [39] (Figs. 4, 21). Surgical management of such tumours can require collaboration between surgeons of different specialties [40].

**Fig. 22:**
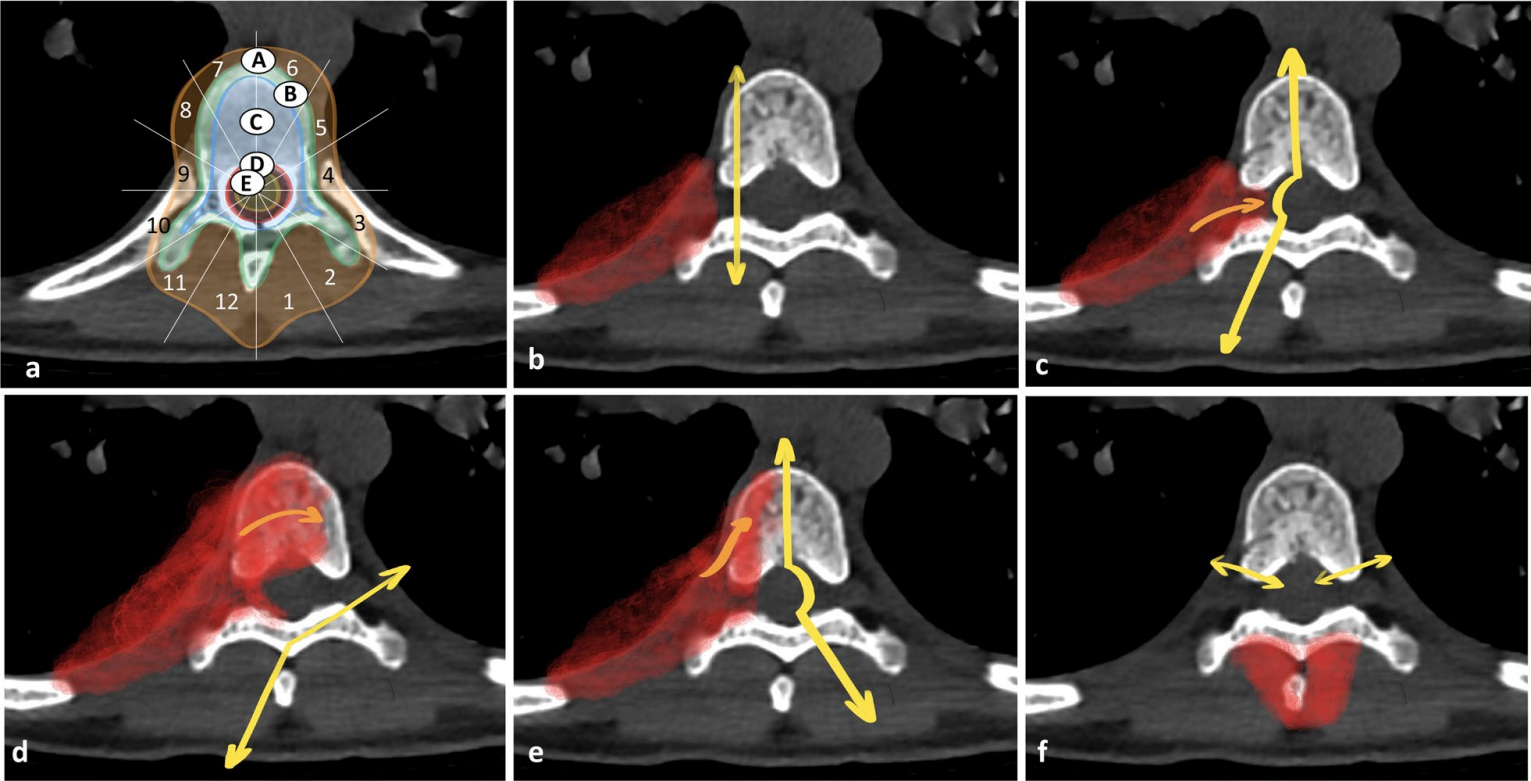
*Weinstein–Boriani–Biagini classification* (**a**). A: paraspinal soft tissues; B: intraosseous (superficial); C: intraosseous (Deep); D: extraosseous (Extradural); E: extraosseous (Intradural). *Fadel and Missenard classification of vertebral osteotomy* (**b**, **c**, **d**, **e**). **b**: transversectomy; **c**: one-third vertebrectomy; **d**: two-third vertebrectomy; **e**: total vertebrectomy. *En bloc* resection of the posterior arch (**f**)

To facilitate treatment planning, axial extension of paraspinal masses can be assessed with the Weinstein–Boriani–Biagini (WBB) classification, which allows oncological staging and treatment guidance (Fig. 22). The WBB classification divides spinal and paraspinal regions into five concentric layers centred on the dural sac and extending up to the paraspinal soft tissues [41]. The classification of Fadel and Missenard, which is usually used to characterise bone extensions from pulmonary masses, can be also used to characterise the soft tissue tumours involving the spine [42]. The structured MRI and CT report for paraspinal lesions is given in Table 3.

**Table 3:** Suggested MRI and CT structured report for paraspinal lesions

MRI reporting
*Size*
Location Epicentre: Epaxial/hypaxial Segmental longitudinal extension
Matrix Fat content (high signal on both T1 and T2WI) Myxoid content (fluid-like signal intensity; enhancement) Fibrous content (low signal on both T1 and T2WI) Haemorrhagic content (signal change with evolving breakdown products; Blooming artefact of T2WI) Necrotic or cystic
Bone involvement (vertebra, rib) Suggestive signs of reactive inflammatory changes or vertebral invasion: cortical abutment and disappearance of paravertebral fat on T1WI, enhancement on T1WI fat-suppressed, periosteal signal change, Bone tumour invasion: low signal on both fat-suppressed T2WI and T1WI, scalloping when present, details the tumour extension within the different parts of the bone
Relationship with spinal nerve and intervertebral foramen Fat plane (high intensity on T1WI) separating the neurovascular pedicle and the tumour Extension into the intervertebral foramen Foramen enlargement
Locoregional invasion of adjacent compartments or organs Pleura Posterior mediastinum Retroperitoneum Epidural space Skip metastasis

Scanner reporting
*Tumour size, location, matrix*
Bone involvement (vertebra, rib) Suggestive of a tumour process: bone lysis (geographics, sclerotic, permeative) Suggestive of mimics: erosions, calcifications, osteodystrophy Foramen enlargement
Distant extension—invasion of others compartments or organs Multiple lesions Invasion to adjacent compartments or organs Lymph nodes status Distant metastatic disease (for malignant and metastasising tumours)

Although certain imaging features can be suggestive of a specific aetiology, pathology reports are often vital to establishing therapeutic strategies. The area and target of image-guided biopsies must therefore be defined through discussion by a multidisciplinary tumour board [21].

## Epidemiology and imaging features of soft tissue tumours of the paraspinal region

The epidemiology of paraspinal soft tissue tumours is not well known; most studies detail paraspinal lesions alongside lesions of the chest wall, abdominal wall and trunk. According to Picci et al., paraspinal tumours represent 3.75% of all soft tissue tumours [43]. The majority of paraspinal soft tissue tumours are benign. However, some reports suggest that paraspinal lesions are proportionally more frequently malignant (from 14.5 to 42%) than lesions in other musculoskeletal system locations, especially for small tumours (< 5 cm) (up to 45.5%) [43, 44]. By far the most frequent benign lesions in the paraspinal region include lipomas, nodular fasciitis, deep fibromatoses and benign PNSTs (BPNSTs) [45]. Among malignant soft tissue tumours, the most represented tumours are undifferentiated pleomorphic sarcomas and liposarcomas. Soft tissue sarcomas in the trunk are associated with a worse prognosis than sarcomas in other locations [46]. We presented here the types that are considered relevant because of their frequency or their typical radiological features, in line with the WHO classification [47]. Table 4 summarises clinical clues, imaging findings and frequency of paraspinal soft tissue tumours.

**Table 4:** Summary of the paraspinal soft tissue tumours discussed in this article with their clinical signs, imaging features and incidence

WHO	Clinical signs	US signs *Method of choice for superficial tumours*	CT signs *Method of choice for calcifications and bone analysis*	MRI signs *Method of choice for soft tissue and bone*	Relative frequency in the back
*Adipocytic tumours*					
Lipoma	40–60 y Mobile Grows with weight gain Multiples (mostly in the back)	Hyperechoic Fine hyperechoic lines Compressible	HU in the negative range	Follows subcutaneous fat signal No nodule No septation No enhancement	Common in upper back
Liposarcoma	40–70 y	Lipoma with atypical features: Hypervascularity Hypoechoic areas	HU in the negative range Calcification	Variable amount of fat content Lipoma with atypical features: nodule/septa /enhancement	Uncommon*
Hibernoma	20–40 y		Attenuation	Flow voids	Exceptional
*PNST*					
BPNST	30–60 y Pain Multiples	–	Foramen enlargement	Dumbbell shape Split-fat sign (T1WI) Tail sign (T1WI) Target sign (T2WI) Fascicular sign (T2WI)	Common
Plexiform neurofibromas	15–30 y Consistency like a «bag of worms» History of NF1	Superficial Multiloculated Cutaneous and subcutaneous Hypoechoic foci	–	Infiltrative appearance Target sign (T2WI)	Rare
MPNST	30–60 y Pain Numbness Paresthesia NF1	–	Foramen enlargement Bone lysis	Large size Heterogeneous signal Perilesional oedema No split-fat sign	Rare
*Fibro/myofibroblastic tumours*					
Desmoid tumour	25–45 y F > M Trauma Rapidly growing Pain Multiples	Fascia based lesion Hypoechoic Staghorn sign Fascial tail sign	–	Tail sign Band sign Temporal change with time hypointense on all sequence	Common
Solitary fibrous tumour	40–60 y	–	Prominent feeding vessels	Prominent feeding vessels Avid enhancement	Rare
Myxofibrosarcoma	50–70 y	–	–	Water-like appearance (T2WI) Tail sign	Rare
*Vascular tumours*					
Haemangioma	20–30 y Painful Physical exertion Fluctuating daily size	Heterogeneous Hypoechoic with multiple cysts Augmentation of out flow by compression Augmentation of in flow by release	Phleboliths	Fat content (T1WI) Serpentine vascular structures Atrophic changes in muscles	Uncommon
Angiosarcoma	60–70 y	–	–	Heterogeneous Vessels (high or low flow) Foci of haemorrhages	Exceptional*
*Myogenic tumours*					
Leiomyoma	–	–	–	–	Exceptional
Rhabdomyosarcoma	< 20 y	–	–	–	Exceptional
Leiomyosarcoma	50–70 y	–	Contiguous with a vessel	Contiguous with a vessel	Rare*
*Chondro-osseous tumours*					
Chondro-osseous tumours	30–60 y	–	Chondroid matrix: arc and ring-like calcifications Bone matrix	Chondroid matrix: High T2 signal	Exceptional
*Tumours with uncertain differentiation*					
Myxoma	30–60 y F > M	Fatty bright cap sign Aligned in muscle fibre axis	–	Fluid-like signal intensity Aligned in muscle fibre axis	Uncommon
Undifferentiated pleomorphic sarcoma	50–75 y	–	–	–	Rare
Synovial sarcoma	15–40 y	–	Calcification	Triple sign (T2WI) Internal haemorrhage	Rare
Muscle metastasis	50–70 y History of cancer	–	Multiple Rim enhancement Central attenuation	Rim enhancement	Common

*Except for retroperitoneal or mediastinal masses with secondary paraspinal invasion

HU, Hounsfield unit; y, years; F, female; M, male; and WI, weighted imaging

### Adipocytic tumours

*Lipomas* represent the most common soft tissue neoplasms [48, 49]. The upper back is among the anatomical regions most frequently affected by lipomas. Imaging features of lipomatous lesions are pathognomonic [48–50]. Lipomas are classically well defined, frequently encapsulated. On US, lipomas are iso to hyperechoic masses within thin transverse hyperechoic lines. On MRI, lipomas present as isointense masses relative to subcutaneous fat, regardless of pulse sequence. Lipomas may present with septa (< 2 mm), with or without enhancement (Fig. 14).

*Liposarcomas*, the malignant counterparts of the lipomas, are the most frequent soft tissue sarcomas [51], are the main differential diagnostic consideration for fat-containing masses in the paraspinal region. The presence of thickened or nodular enhancing septa with fat attenuation on CT images or fat signal intensity with MRI as well as hypervascularity and hypoechoic areas on US should raise concern for liposarcoma (Fig. 3) [52].

A few cases of *hibernomas* have been described in the paraspinal region [53].

### Peripheral nerve sheath tumours (PNST)

The spine is surrounded by nerves of various origins; consequently, various types of neurogenic tumours can affect the paraspinal region [54, 55].

*Benign peripheral nerve sheath tumours* are classified into schwannomas and neurofibromas. Around 15% of the PNST are axial, their majority being present in either the brachial or lumbosacral plexus [56]. Neurofibroma are more than twice as common in the trunk than schwannoma [55]. Classically, they present as fusiform masses along a nerve that depicts the ‘tail sign’. Paraspinal BPNSTs, arising from the spinal nerve or its roots, may have a typical dumbbell configuration, with intraspinal and paraspinal components that communicate via an intervertebral foramen (Fig. 17). They demonstrate low signal intensity on T1WI and high signal intensity on T2WI, sometimes with a distinctive central hypointense signal surrounded by hyperintense signals on T2WI termed the ‘target sign’. A ring of fat, observed over the proximal and distal poles of masses, the ‘split-fat sign’, is highly suggestive of neural neoplasms. The fascicular sign, seen on T2WI, characterised by multiple small ring-like structures with peripheral hyperintensity (the fascicular bundles) suggests a lesion of neurogenic origin. Older tumours may contain cystic degeneration and calcifications along the walls of masses (Fig. 18). BPNSTs usually demonstrate contrast enhancement [57]. Plexiform neurofibromas are neurofibroma variants that affect nerves in patients with neurofibromatosis type 1 (Additional file 1: Fig. S2) and have a large lobulated or infiltrative appearance [57].

*Malignant peripheral nerve sheath tumours* (MPNSTs) are rare (10% of the PNST) but are more common where there is history of spinal irradiation. Radiological features of MPNSTs are non-specific; however, four imaging findings are suggestive of the diagnosis: a large size (> 5 cm), infiltrative margins with perilesional oedema, absence of split-fat sign and heterogeneous signals on T1WI, T2WI and postcontrast images [28, 54].

### Fibroblastic/myofibroblastic tumours

Fibrous soft tissue tumours are grouped into three main categories: *benign fibrous proliferations*, *fibromatoses* and *fibrosarcomas* [58]. Fibroblastic tumours typically present a fascial tail sign. Features of benign fibroblastic tumours on MRI relates to the ratio between cellularity and collagen content. Young hypercellular tumours are almost isointense to skeletal muscle in T1WI and hyperintense to adipose tissue in T2WI. As fibroblastic tumours evolve, collagen deposition increases, cellularity decreases and tumours become hypointense in all sequences.

*Nodular fasciitis* is a benign proliferation of myofibroblasts and fibroblasts [58]. The trunk is the second most frequent location that nodular fasciitis occurs in; around 20% of all cases occur in this location. Nodular fasciitis (either subcutaneous, intramuscular or fascial) usually manifests as small (< 4 cm) subcutaneous, fascia-based and circumscribed tumours.

*Deep (or musculoaponeurotic or extraabdominal) fibromatoses*, also called desmoid tumours, originate from the connective tissue in muscle, fasciae or aponeuroses. Desmoid tumours extend along muscles and fasciae. In US, paraspinal desmoid tumours present as oval, large (> 5 cm), deep solid masses with smooth or poor margins and variable echogenicity. Local invasion along fibrous septa into the subcutaneous fat resembles a ‘branching staghorn’ [59]. On MRI, desmoid tumours are characterised the presence of hypointense and unenhanced collagenous bands surrounded by cellular regions of higher signal intensity on T2WI, termed the ‘band sign’ (Fig. 15).

*Myxofibrosarcoma*, the most frequent subtype of malignant fibroblastic tumours, is located in the trunk in 8 to 11% of all cases [60]. Up to 75% of myxofibrosarcomas are superficial. High myxoid matrix content is responsible for a water-like appearance on fluid-sensitive sequences [61].

### Vascular tumours

*Haemangiomas* are common neoplasms of a benign histological origin. They contain large serpentine vessels, adjacent stromal fatty proliferation and phleboliths [49, 62]. MRI features are usually characteristic and demonstrate areas of high signal intensity on T1WI corresponding to internal fat and high signal intensity on T2WI in the central angiomatous core of the mass (Fig. 15) [62, 63]. Serpentine vascular structures are seen either with contrast enhancement or as flow voids if blood flow is rapid enough. Remodelling of the bone in response to changes in local vascular flow may also be present and best seen on CT.

One case of *malignant vascular tumour* in the thoracic paraspinal muscles has been reported in the literature [64].

### Myogenic tumours

**Fig. 23:**
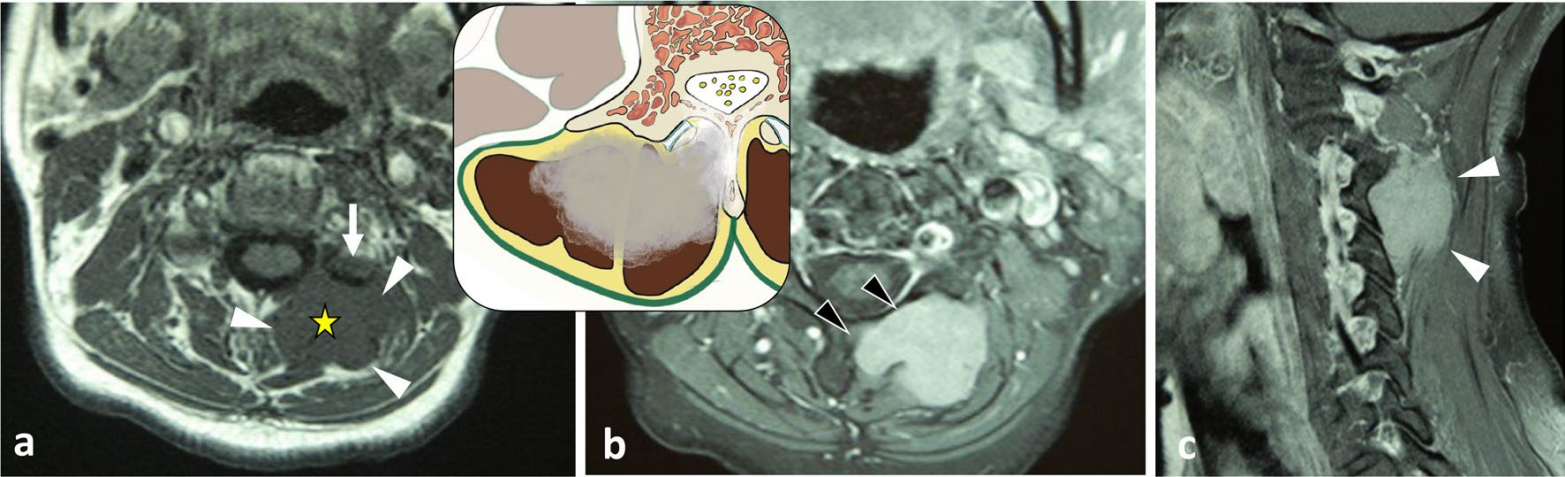
*Leiomyoma* in a 60-year-old male with non-mechanical cervical pain. MRI scan shows a lobulated mass in left epaxial muscles (white arrowheads) with low signal intensity on T1WI (**a**) and enhancement on axial (**b**) and sagittal (**c**) contrast-enhanced fat-saturated T1WI. Several features are suggestive of vertebral bone invasion: disappearance of the fat plane separating the mass and the neural arch associated with a low signal of medullary bone of the articular process on T1WI (white arrow), disappearance of cortical bone and enhancement of medullary bone (black arrowheads) on contrast-enhanced T1WI. The yellow star shows the epicentre of the mass

Benign and malignant soft tissue tumours with muscle differentiation are extremely rare, even more so in the paraspinal region. Only a few case reports of *leiomyomas* and *rhabdomyomas* of the paraspinal region have been described [65–68]. They present with the same features as those described in other locations (Fig. 23).

*Somatic leiomyosarcoma* is one of the three most common soft tissue sarcomas. Its occurrence in an epaxial location is extremely rare (2% of cases) [69]. Instead, retroperitoneum is a common site of origin, accounting for 12%–69% of cases. Given the absence of anatomical barrier between the paraspinal region and the retroperitoneum, leiomyosarcomas often invade the hypaxial muscles [70]. Imaging features of leiomyosarcomas are non-specific; they display heterogeneous hypointensity on T1WI, with a thick, irregular rim enhancement on contrast T1, and high signal intensity on T2WI (Fig. 7). Seven per cent of *rhabdomyosarcoma* are found in the trunk.

### Chondro-osseous tumours

**Fig. 24:**
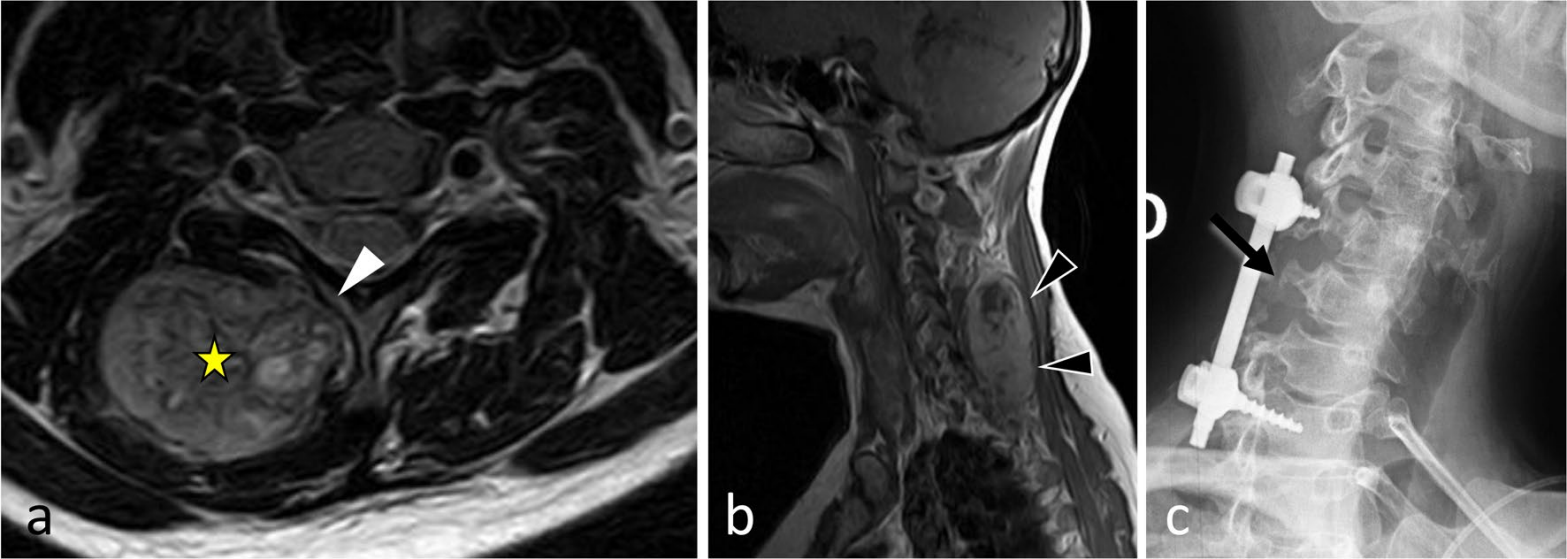
*Mesenchymal chondrosarcoma* of right hypaxial muscles in a 37-year-old female with chronic cervical pain and bump in the neck. MRI shows a lobulated mass with high signal on axial T2WI (**a**) and heterogeneous enhancement pattern after contrast injection (black arrowheads, **b**). White arrowhead demonstrates the disappearance of the fat plane separating the mass and the neural arch. The yellow star shows the epicentre of the mass. Cervical radiograph (oblique view) shows posterior cervical fixation following laminectomy (**c**)

Soft tissue chondromas and extraskeletal osteosarcomas represent exceedingly rare lesions, and occur almost exclusively in the extremities. The few reports of extraskeletal soft tissue chondromas and extraskeletal osteosarcomas are the evidence of existence of these tumours in the paraspinal region [71]. Figure 24 depicts a case of *mesenchymal chondrosarcoma* in the cervical epaxial region that was treated in our centre.

### Tumours with uncertain differentiation

*Myxomas* are rare soft tissue tumours and occurs in the spine in less than 10% of cases [72]. Myxomas are painful in around half of cases. Intramuscular myxomas present as well-defined, ovoid masses aligned in the muscle fibre axis [73, 74]. A triangular hyperechoic area (also seen as a high signal intensity on MRI), named the fatty ‘bright cap sign’, is seen adjacent to at least one of the poles of the mass. Myxomas exhibit homogeneous fluid-like signal intensity with variable enhancement.

*Undifferentiated sarcomas* are one of the three most frequent soft tissue sarcomas. The trunk encompasses less than 10% of undifferentiated sarcomas [75]. Like most soft tissue sarcomas, undifferentiated sarcomas have non-specific imaging features [76]. Undifferentiated sarcomas usually present as large and well-circumscribed masses with intermediate signal intensity on T1WI and high signal intensity on T2WI (Fig. 21). A ‘tail sign’ can be present, as can calcifications and myxoid content [77].

**Fig. 25:**
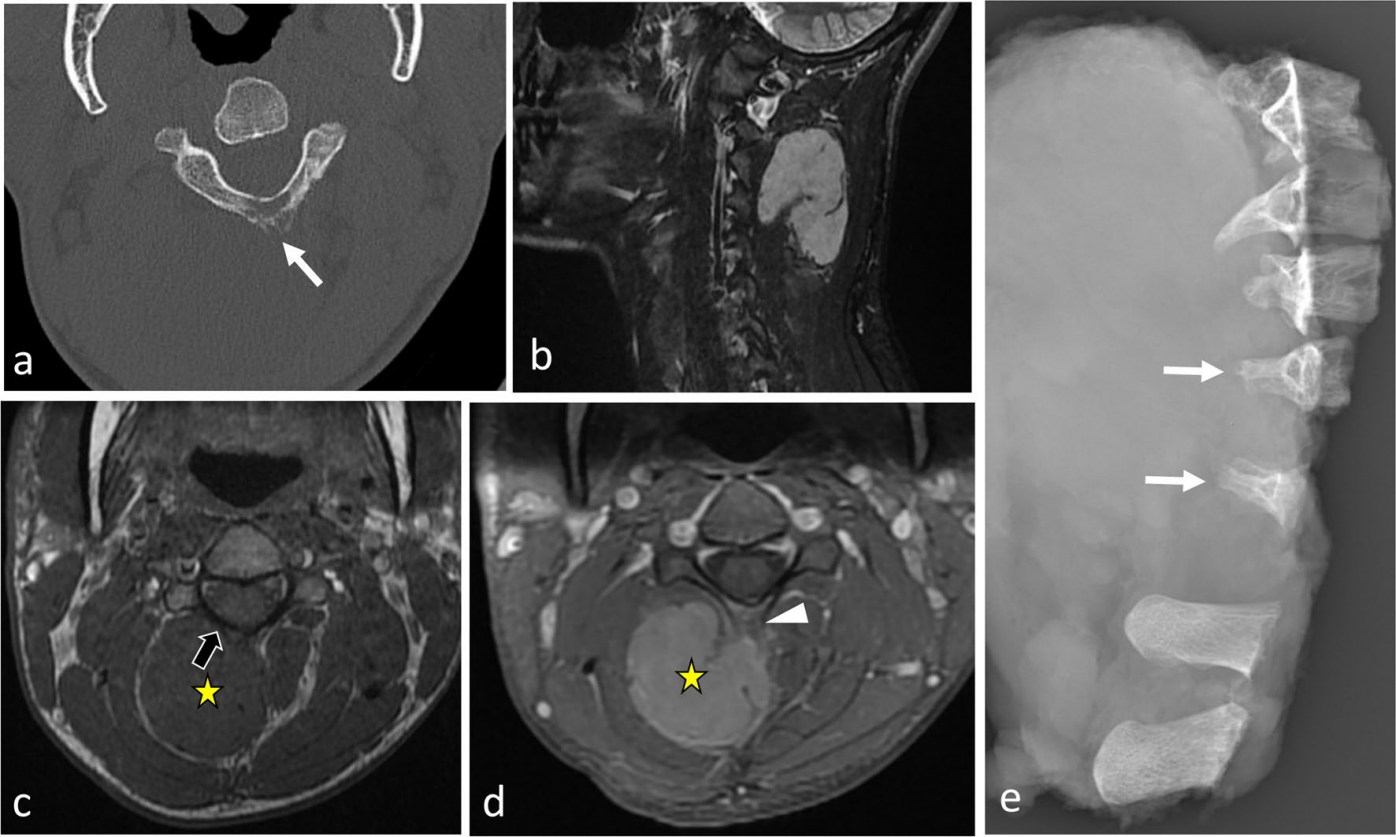
*Synovial sarcoma* of deep epaxial muscles at C4 level in a 53-year-old male with a bump and acute non-mechanical cervical pain. The mass presents aggressive radiological features such as cortical and trabecular lysis of the neural arch of C4 (**a**) with moth-eaten margin. The mass has low intensity on T1WI (**b**), with enhancement on contrast-enhanced fat-suppressed T1WI (**c**) and high signal on T2WI (**d**). MRI features suggestive of bone invasion were: the disappearance of the fat plane separating the mass and the lamina (black arrow, **c**) the enhancement of medullary bone of the spinous process (white arrowhead, **d**) and the enhancement of periosteum of right lamina (black arrowhead, **d**). Lateral radiograph of the surgical sample (two-third vertebrectomy) (**e**) shows bone lysis of the tip of the vertebral processes (C4, C5 and C6) (white arrows). The yellow star shows the epicentre of the mass.

*Synovial sarcomas* are very rare in the spine [78]. Two-thirds of synovial sarcomas are found in the extremities, while less than 2.4 to 7% are located in the spine, more frequently in the cervical and thoracic spine than in the lumbar spine [79]. CT images show a heterogeneous mass with frequent areas of haemorrhage and necrosis, along with cystic areas and peripheral calcifications (Fig. 25) [80]. MRI typically shows a heterogeneous multinodular soft tissue mass. T2WI depict the typical—but non-specific—‘triple sign’ composed of intermixed areas of low, intermediate and high signal intensity.

### Muscle metastases

**Fig. 26:**
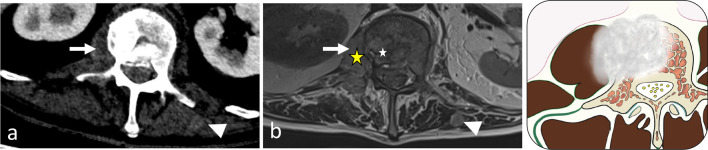
*Muscle and bone metastasis* from a lung cancer in a 70-year-old male with acute back pain. Contrast-enhanced axial CT scan reconstruction (**a**) and axial contrast-enhanced T1WI (**b**) show a spinal metastasis extending into the psoas muscle and the epaxial region (arrow) and an intramuscular metastasis (arrowhead). The yellow star shows the epicentre of the mass

Until recently, skeletal muscle metastases were considered to be rare entities [81, 82]. With the advent of positron emission tomography (PET)/CT, its diagnosis has improved, and it appears to occur more frequently than previously suggested. The most common primaries involved are lung, urogenital and gastrointestinal systems cancers. Trunk muscles are the most frequent sites of muscle metastases and account for 27.8 to 49% of all of the muscle metastases. Muscle metastasis locates particularly in the iliopsoas (13.6%), the erector spinae (4.9) and the paraspinal muscles (2.8%). The CT appearance of muscle metastasis is usually a ring-shaped pattern with focal areas of central necrosis [83]. Muscle metastases present with low to intermediate signal intensity on T1WI and high signal intensity on T2WI, with a pattern of rim enhancement (Fig. 26). Less frequently, it corresponds to a diffuse metastatic muscle infiltration or a focal intramuscular mass with homogeneous contrast enhancement lesion.

## Epidemiology and imaging features of spinal bone tumours with prominent paraspinal extension

**Fig. 27:**
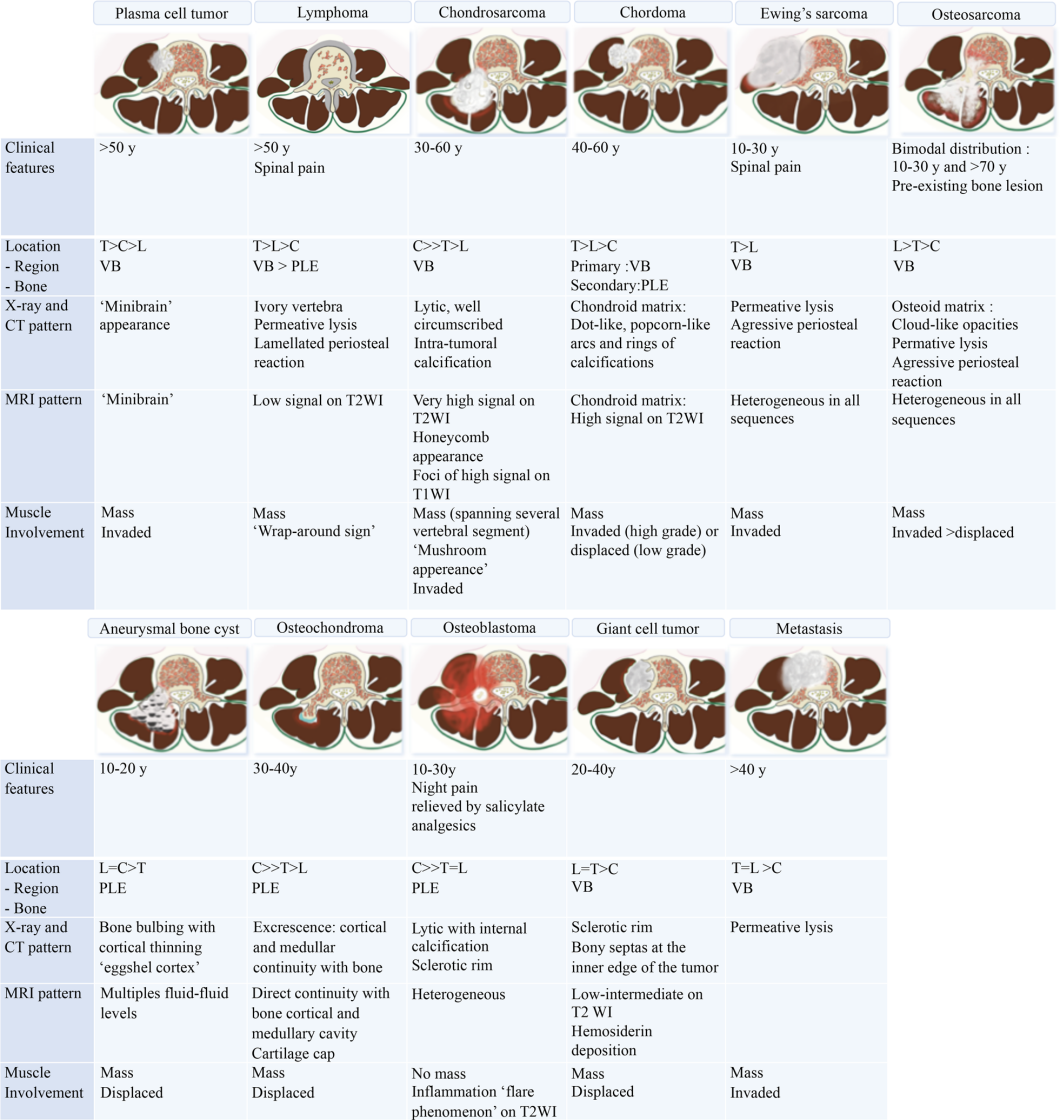
*Summary of clinical features and imaging patterns for expansive spinal tumours with focus on paraspinal muscle involvement*. C, cervical spine; L, lumbar spine; T, thoracic spine; VB, vertebral body; and PLE, posterolateral elements

Certain spinal tumours may present as midline expansile masses, in which the extraosseous component of the tumour—either displacing or invading the adjacent paraspinal musculature—is often much larger than the bone lesion itself [84]. In spinal tumour classification, these tumours are recognised as ‘extracompartmental’ spinal tumours; ‘intracompartmental’ tumours are confined to the bone. In contrast to paraspinal tumours originating from soft tissues, paraspinal masses from spinal elements are always associated with vertebral bone changes (bone production or bone destruction). In most cases, in contrast to paraspinal masses originating from soft tissue, an expansile spinal lesion is limited to one vertebral segment; however, certain tumours, including osteochondromas, chordomas, giant cell tumours and aneurismal bone cysts, may infiltrate the intervertebral disc space and spread to contiguous vertebrae. Epidemiology is a key element for differential diagnosis of expansive vertebral tumour [85]. Expansile bone lesions of vertebrae are usually classified as benign locally aggressive or malignant. In most cases, radiological features did not allow a differential diagnosis between a benign lesion, a primary tumour, or a metastasis. Here, we discuss only exophytic spinal tumours that exhibit specific imaging features and for which paraspinal involvement. Figure 27 summarises clinical signs, anatomical location and imaging findings of exophytic spinal tumours.

### Benign locally aggressive spinal tumours

**Fig. 28:**
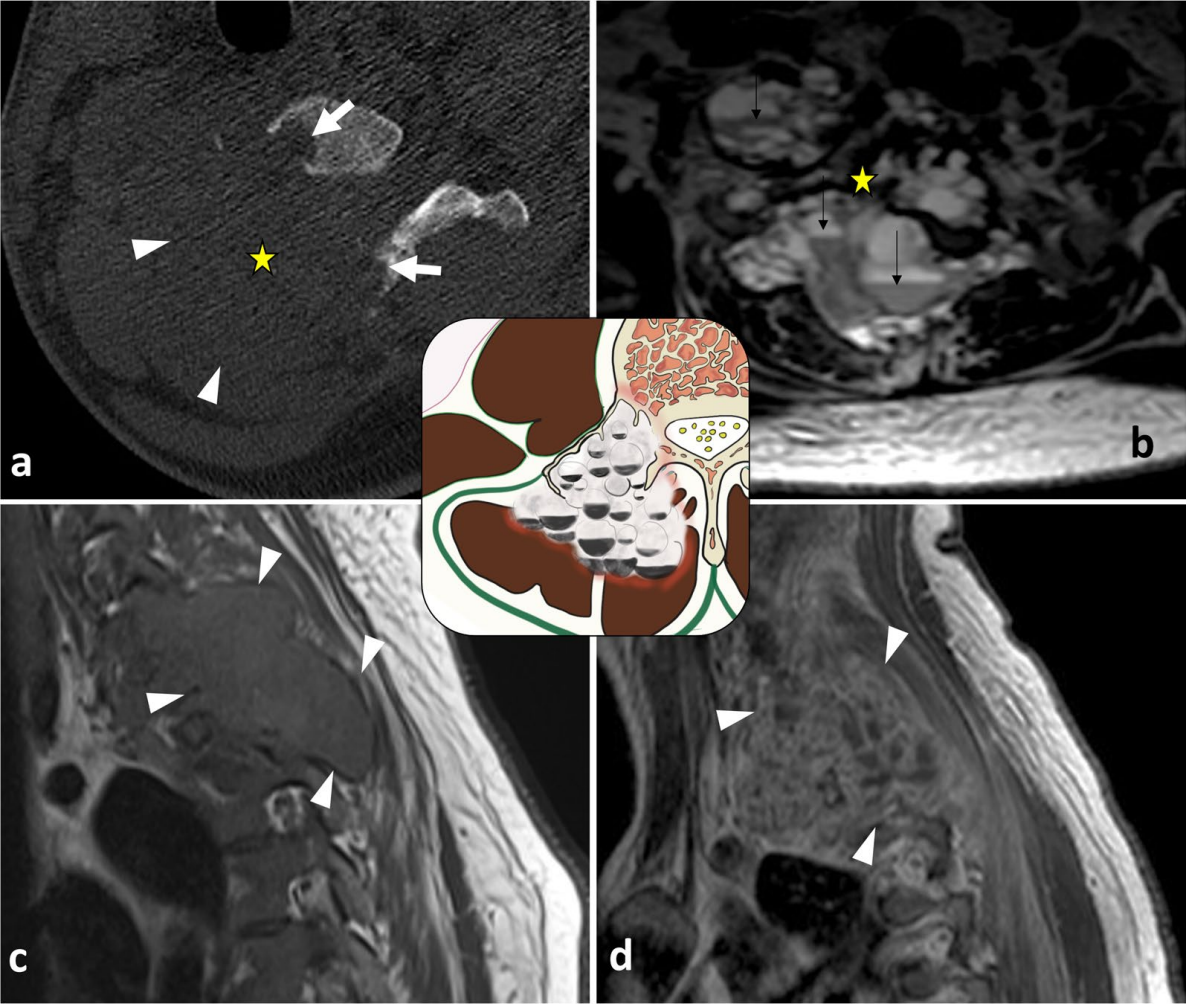
*Aneurysmal bone cyst* in a 27-year-old female with cervical pain and weakness of the right upper limb. Axial CT scan reconstruction (**a**) shows an expansile, lytic lesion within the right neural arch and the vertebral body of C6 (white arrows). The mass invades epaxial and hypaxial region (arrowheads). Axial T2WI (**b**) demonstrates fluid–fluid levels (black arrows). Sagittal T1WI (**c**) and contrast-enhanced T1WI (**d**) show cystic areas. The lesion involves two adjacent vertebral levels. The yellow star shows the epicentre of the mass

*Aneurysmal bone cysts (ABCs)* are a type of neoplasm thought to arise from local circulatory disturbance and are typically located in the spine [86]. Most ABCs are primary, although one-third are secondary to another lesion. CT imaging reveals osteolytic, eccentric and multiloculated lesions with well-defined—sometimes sclerotic—margins [87]. Spinal ABCs lead to bone bulging with typical cortical thinning (‘eggshell cortex’), which is best seen on CT images (Fig. 28). ABCs often extend into the paravertebral soft tissues. On MRI, ABCs appear as lobular lesions with septa and multiple fluid–fluid levels. Peripheral, septal or nodular enhancement is observed.

**Fig. 29:**
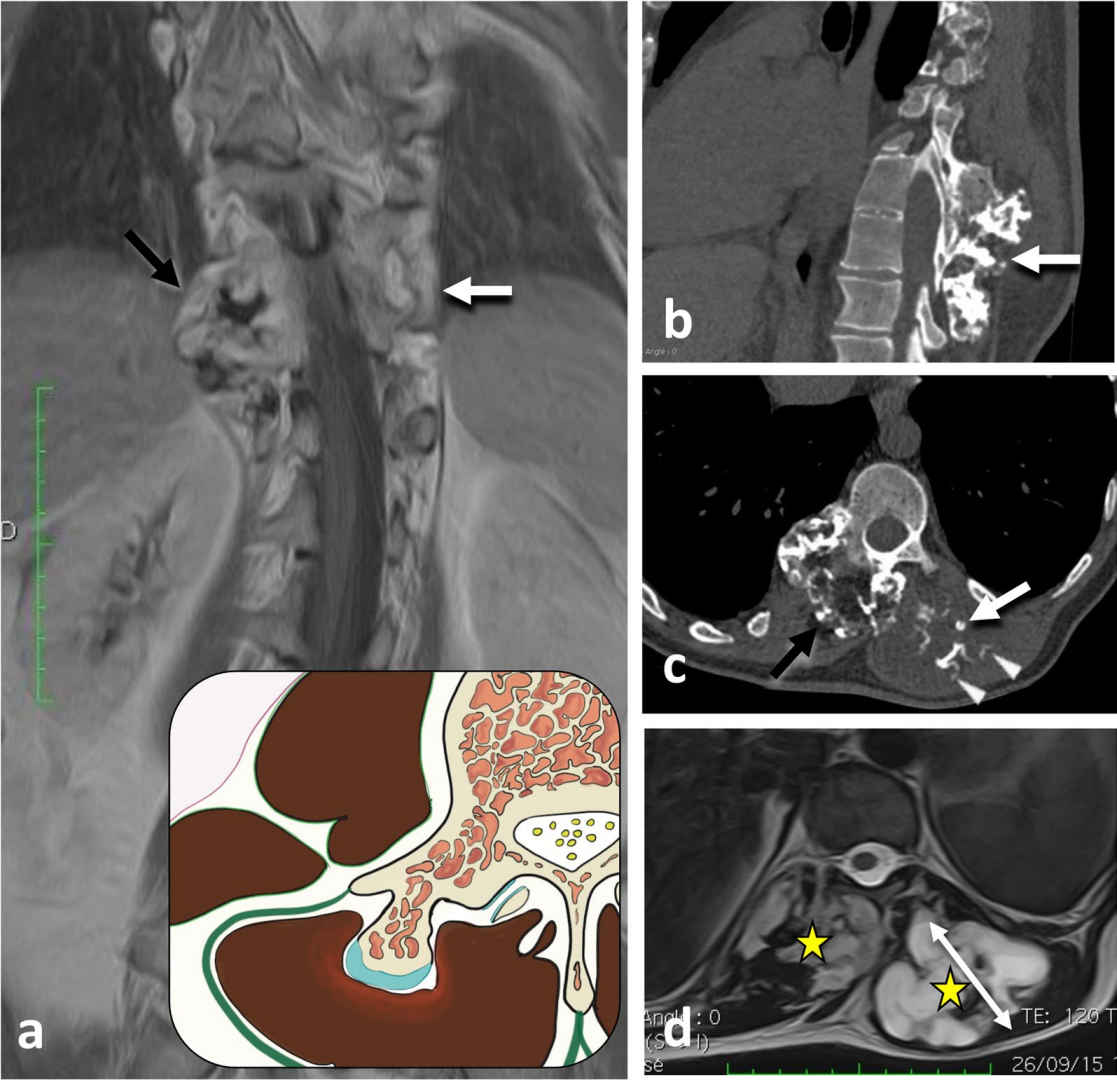
*Exostosis and chondrosarcoma*, respectively, on the right and on the left transverse processes of T10 in a 57-year-old male with hereditary multiple exostosis. The patient had been experiencing back pain for 2 months, a possible indicator of malignant degeneration of a chondroma into a chondrosarcoma. The patient presents with a right osseous outgrowth (black arrows), which has cortical and medullary continuity with the underlying bone as shown in coronal T1WI (**a**) and axial CT scan reconstruction (**c**). The cartilage cap of both exostosis and chondrosarcoma appears with intermediate signal on T1WI (**a**) and high signal on T2WI (**d**) and has ring and arc calcifications (arrowheads). The patient presents a left osseous outgrowth (white arrows), with rough cortex and cortical disruption (**b**, **c**) and with thick cartilage cap (3 cm, double arrow) on axial T2WI (**d**). Yellow stars show the epicentre of masses

*Osteochondromas,* or *exostoses,* are cartilage-forming tumours. Osteochondromas are the most common bone tumours but osteochondromas of the spine are a relatively rare [88, 89]. Osteochondromas are growths of bone extending outwards from the surface of vertebrae with direct continuity with the cortical and medullary cavity of the underlying bone; they often arise from the rib head [89]. A cartilage cap with a similar appearance to normal cartilage covers the lesion (Fig. 29). MRI allows cartilage thickness to be assessed; when this is > 1.5 cm, this suggests a sarcomatous transformation.

*Osteoblastomas* are bone-forming tumours with a particular predilection for the spine. Spinal osteoblastomas form one-third of all osteoblastomas [90]. On CT imaging, osteoblastomas typically appear as lytic expansile lesions with internal calcifications and a sclerotic rim [91]. Less frequently, they can exhibit a bubbly appearance, or an aggressive appearance without sclerotic margins and can be heavily calcified (Fig. 12) [92]. MRI features of the lesion tend to be non-specific, with hypo- to isointensity on T1WI and hypo- to hyperintensity on T2WI and areas of decreased intensity related to calcifications. MRI typically exhibits significant peritumoural bone inflammation and paraspinal soft tissue inflammation with hyperintensity on T2WI and high enhancement after contrast administration, referred to as the ‘flare phenomenon’ [92]. A discrepancy between the localised tumour observed on CT images and the huge reactive changes in the paraspinal soft tissues observed on MRI is strongly suggestive of osteoblastoma.

*Chordomas* are bone tumours derived from embryological remnants of the notochord [93]**.** Typical radiological features consist of lytic bone destruction and a disproportionately large soft tissue mass with a ‘mushroom appearance’. Calcifications, septa and internal matrix mineralisation are frequent. MRI shows predominant high intensity on T2WI, hypointensity on T1WI and heterogeneous enhancement.

**Fig. 30:**
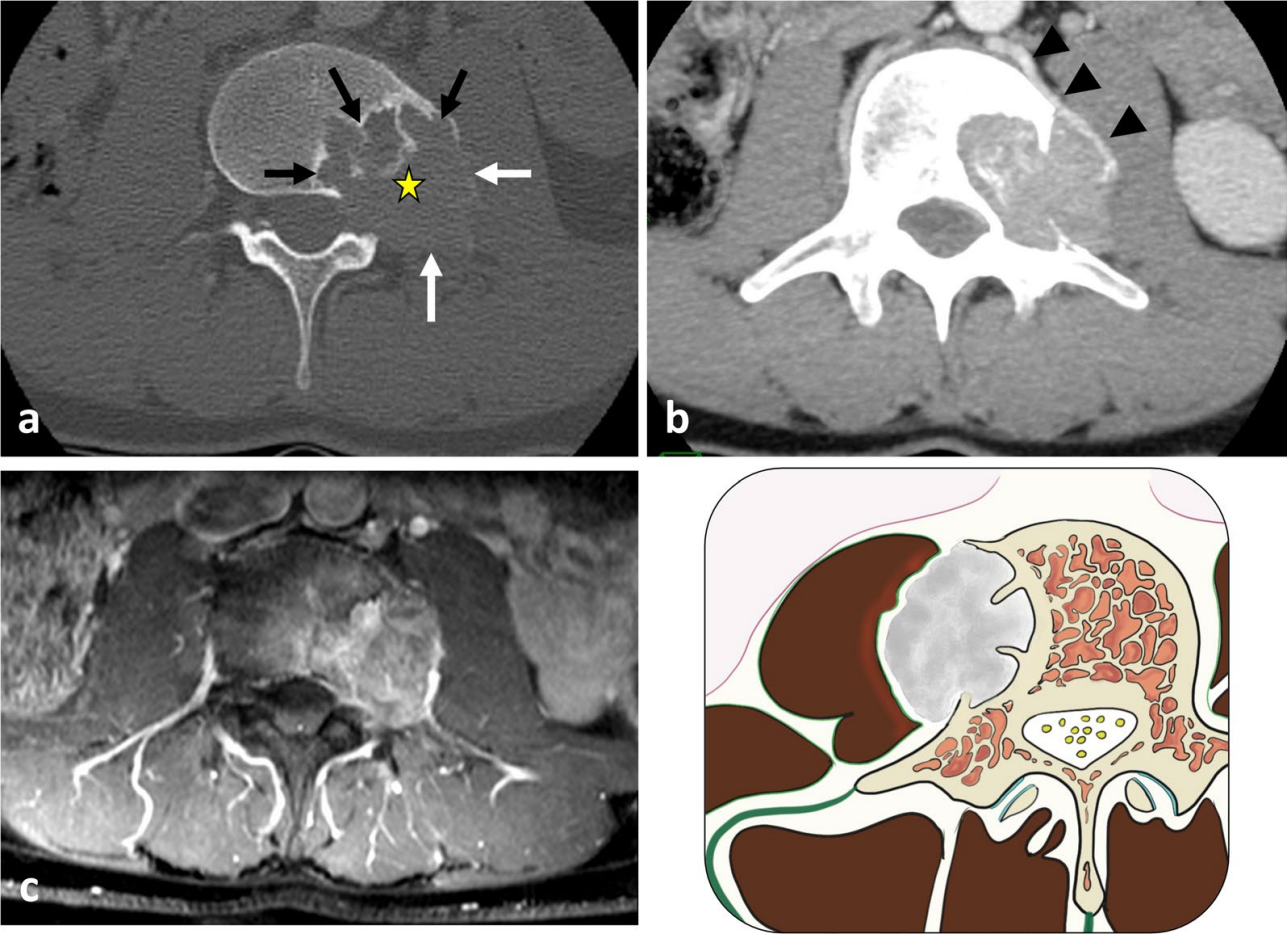
*Giant cell tumour* with left corporeal-pedicle development in a 42-year-old female with low back pain of 4 months of duration (**a**, **b**, **c**). Axial CT scan reconstruction (**a**) shows a lytic lesion with a sclerotic rim (black arrow) at the border of the tumour opposite to the eccentric side. As shown on axial CT with contrast agent (**b**), the tumour typically splays rather than engulfs the psoas muscles (white arrows) and the lumbar artery (arrowheads). Axial gadolinium-enhanced T1WI (**c**) demonstrates homogeneous and moderate enhancement. Yellow star shows the epicentre of the mass

*Giant cell tumours (GCTs)* are characterised by abundant multinucleated, osteoclastic giant cells [94]. Spinal GCTs extend to the paravertebral soft tissues in almost two-thirds of cases. On radiographs and CT imaging, GCTs are expansile, lytic, eccentric lesions without calcification [94]. A sclerotic rim and bony septa may appear at the border of the tumour that extends to one side of the centre of the vertebra (Fig. 30). T2WI typically reveals a low-signal-intensity lesion [95]. GCTs can contain necrosis and haemorrhages with fluid–fluid levels or cystic areas with high intensity on T1WI and T2WI.

### Malignant spinal tumours

By some distance, spinal metastases are the most frequent malignant neoplasms of the spine, followed by plasma cell tumours [96]. Primary spinal tumours are rare, making up 10% of all spinal neoplasms. Among them, osteosarcomas are the most frequent, followed by chondrosarcomas, Ewing sarcomas and chordomas.

*Metastasis* are frequent [96]. Most spinal metastases originate from breast, lung or prostate cancer. An extracompartmental tumour with paraspinal extension is not the most common presentation of a metastatic spinal tumour [97]. However, given that metastasis occurs more frequently than other spinal tumours, it should always be considered for any extracompartmental spinal tumour cases. Imaging features of spinal metastasis are non-specific; these are lytic lesions with high T2 signal, low T1 signal and contrast enhancement (Figs. 4, 25).

*Plasma cell tumours* are characterised by an accumulation of monoclonal plasma cells and occur in adults, presenting as multiple myelomas or solitary plasmacytomas. Some solitary spinal extradural plasmacytomas involving mainly the paraspinal region have been described [98]. On CT and MRI, plasmacytomas present as lytic bone lesions with sclerosis of the outer cortex and bony septa, resulting in a ‘mini-brain’ appearance [20].

**Fig. 31:**
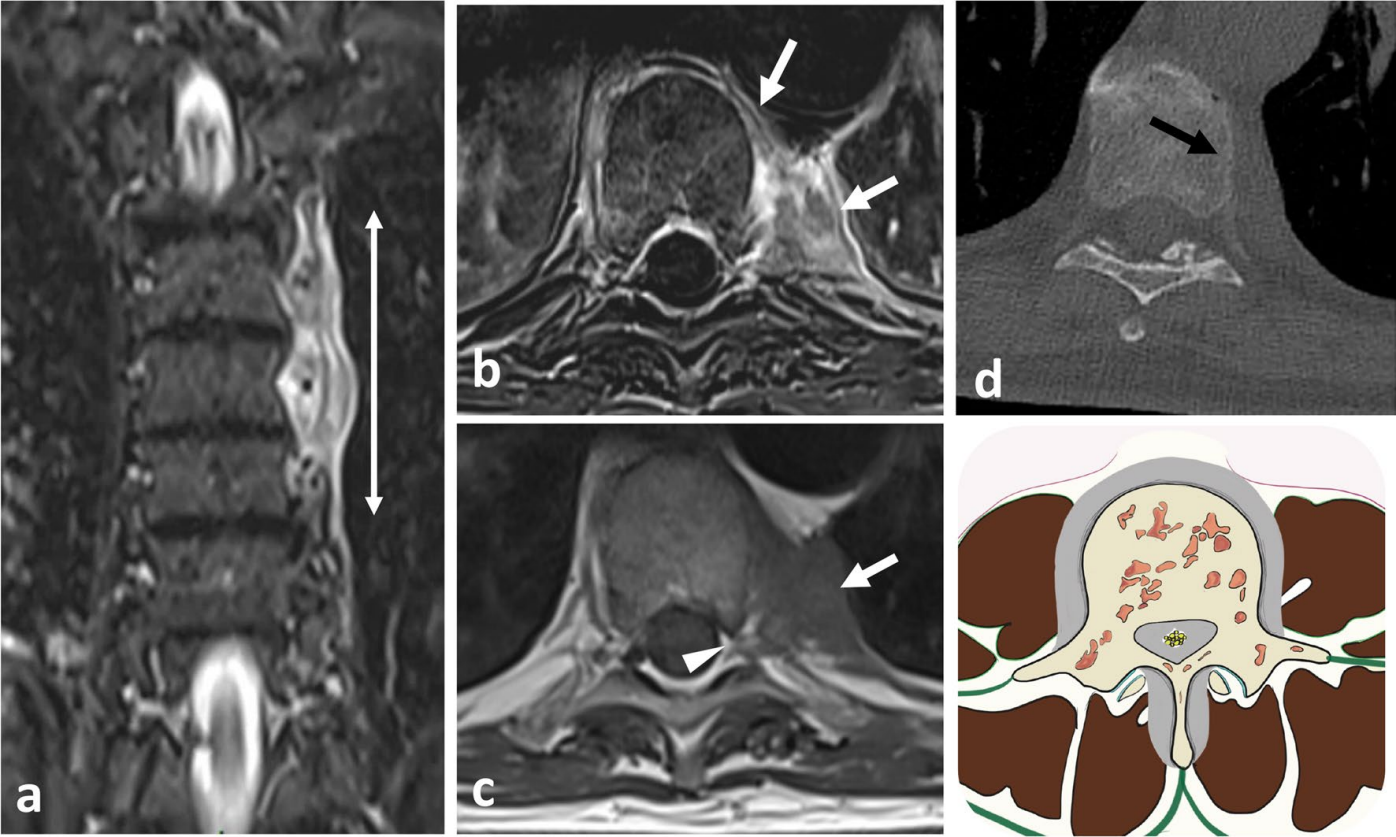
*Non-Hodgkin lymphoma* in a 72-year-old male with fatigue and mild thoracic pain at rest. Coronal STIR (**a**) shows left paraspinal infiltrative mass along three vertebral segments (double head arrow). Axial T1WI (**b**) and subtraction T1WI (**c**) shows an epaxial mass (white arrows), which extents on the ventral surface of the vertebral body and penetrates into the vertebral foramen (arrowhead). Both MRI and CT (**d**) demonstrate that the mass do not invades the vertebra (black arrow)

*Vertebral lymphoma* with paraspinal extent can be primary or secondary, associated with a more widespread systemic disease. Vertebral lymphoma typically displays a circumferential paravertebral involvement termed the ‘wrap-around sign’, and preserved depiction of the intervening vertebral cortex [99]. The wrap-around sign commonly involves the vertebral body but might also be limited only to the posterior elements. The paraspinal mass usually affects several spinal levels sparing the intervertebral disc (Fig. 31). Epidural encasement of the spinal cord is almost always associated. On CT, vertebral lymphoma demonstrates either a permeative pattern or a homogeneous sclerosis of a vertebral body with an ‘ivory vertebra’ appearance [99]. Lamellated pattern of periosteal reaction is possible. On MRI, the paraspinal involvement may have isointense to mildly hypointense T2WI signal, reflecting hypercellularity.

*Chondrosarcomas* are slow-growing, malignant chondrogenic tumours [100]. They are generally primary but can be secondary to a malignant transformation of underlying enchondromas or osteochondromas. Chondrosarcomas of the spine usually manifest as lobulated lytic lesions with soft tissue masses containing flocculent calcifications (Figs. 10, 28). Chondrosarcomas exhibit the characteristic features of the cartilage matrix: the so-called dot-like, popcorn-like arcs and rings of calcifications seen on CT and high intensity signal on T2WI [101].

**Fig. 32:**
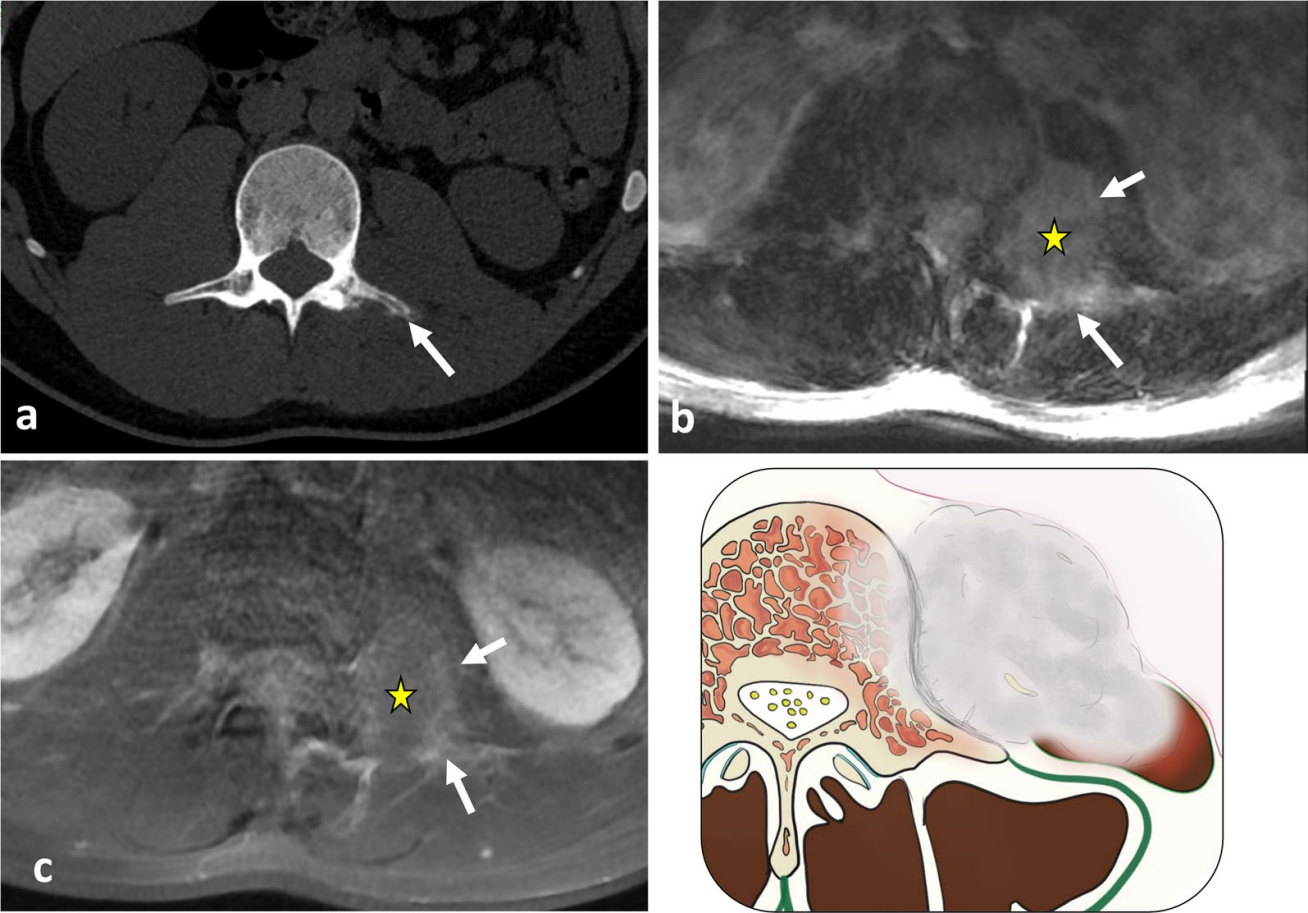
*Ewing sarcoma* of L4 in an 18-year-old male with extreme low back pain and acute cauda equina syndrome. Axial CT reconstruction (**a**) demonstrates a moth-eaten lytic lesion of the transverse process with large paraspinal soft tissue component, best seen on axial T2WI (**b**) and axial contrast-enhanced, fat-suppressed T1WI (**c**). Yellow star shows the epicentre of the mass. Lying position in MRI tube caused intense spinal pain, which explains motion of the patient and motion artefacts

*Vertebral Ewing sarcoma* is a small round blue cell tumour [102]. Spinal Ewing sarcomas present as moth-eaten and permeative lytic lesions with a large paraspinal soft tissue component. The paraspinal mass is often larger than the osseous one. Lamellated periosteal reactions are common [103]. On MRI, Ewing sarcomas often present as heterogeneous tumours with haemorrhage, necrosis and calcifications (Figs. 4, 32).

*Vertebral osteosarcomas* are fast-growing tumours producing osteoid and immature bone [104]. Osteosarcomas are either primary, or secondary, developing within a pre-existing benign bone lesion such as Paget’s disease or after radiotherapy. Among various histological types of osteosarcomas, the most frequent is the osteoblastic or conventional type [102]. Vertebral osteosarcomas arise from the posterior elements with some involvement of vertebral bodies. On CT imaging, conventional osteosarcomas exhibit an osteoid matrix with neoplastic bone formation in the form of cloud-like opacities, associated with various degrees with permeative and destructive processes (Fig. 11). High levels of biological activity are associated with aggressive periosteal reactions and heterogeneous soft tissue masses.

## Paraspinal tumour-like lesions

Paraspinal tumour-like lesions are more common than primary paraspinal and spinal neoplasms. Where imaging, medical history and biological assessments are in agreement, traumatic disorders, degenerative disorders, infections and extramedullary haematopoiesis (EMH) can be easily diagnosed. Table 5 summarises clinical and imaging findings of paraspinal tumours mimics.

**Table 5:** Summary of the paraspinal tumours-like lesions discussed in this article with their clinical and imaging features

Tumour mimics	Notable clinical signs	US (*For superficial masses*)	Notable CT pattern	Notable MRI pattern
Traumatic disorders	History of trauma	*Haematoma:* Compressible fluid collection Low echogenicity Septa *Fat necrosis:* Compressible fluid collection *Morel lavallée:* Compressible fluid collection	*Haematoma:* Hyperdensity	*Haematoma:* Signal changes on T1 and T2 with evolving breakdown products Low signal on T2WI Blooming artefact of T2WI *Fat necrosis:* Fibrous tissue (low signal) intermixed with adipose tissue (high signal) Peripheral enhancement *Morel lavallée:* Fluid collection
Degenerative disorders	> 50 y Spinal degenerative disorders Lumbar predominance	–	Degenerative features: Vertebral narrowing, facet hypertrophy, sclerosis, osteophyte	*Bursitis*: fluid-like signal *Cystic formation* *Fibrous nodules:* Low signal on all sequences
Infection	Increase with age	–	Erosive facet joint Erosive vertebral endplate	*Inflammation:* Imaging psoas sign *Abscess:* Rim enhancement Central attenuation
HEM	Myeloproliferative neoplasm Chronic haematological disorders	–	Multifocal—bilateral Skeletal changes related with chronic anaemia	Smoothy loculated Homogeneous signal Fatty replacement and iron deposition in older masses
MOC	History of trauma Repeated minor injuries Rapidly growing painful	–	Peripheral zonal ossification	Change with time

y, years; WI, weighted imaging

### Traumatic disorders

The back, and especially the lower back, is subject to direct trauma. Diagnosis of a traumatic lesion is easy when there is a history of a traumatic event ahead of the appearance of a painful soft tissue mass with lesions on the overlying skin. In contrast, diagnosis becomes challenging when the interval between the traumatic event and observation of a palpable lump is prolonged; trauma is often underreported. Several types of lesions can occur.

**Fig. 33:**
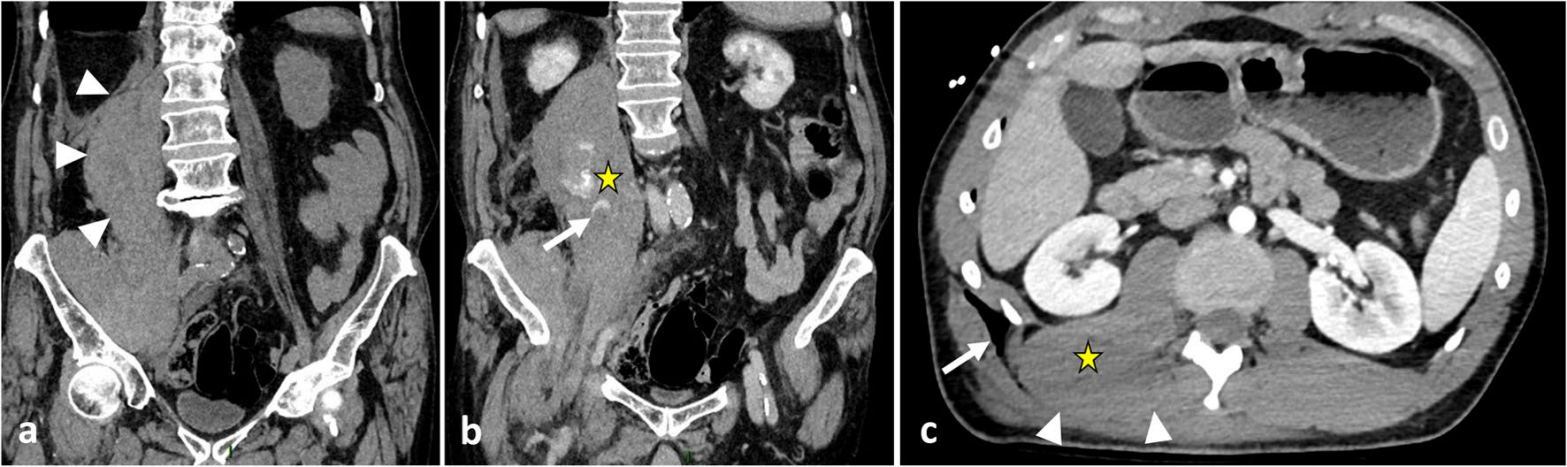
*Trauma conditions* examples in in the paraspinal region. Right *psoas haematoma* in a 76-year-old female following a fall. Coronal CT scan reconstruction without (**a**) and with contrast agent (**b**) shows enlarged right psoas (arrowheads) with active extravasation of contrast (arrow). Muscle *contusion of the right erector spinae muscle* in a 27-year-old male after bicycle accident (**c**). Contrast-enhanced axial CT scan reconstruction (**c**) demonstrates enlarged erector spinae muscle (arrowheads) and subcutaneous emphysema (arrow). Yellow stars show epicentre of masses

*Haematomas* are the most frequent complication of soft tissue injury and exhibit characteristic features in all imaging modalities (Fig. 33). US demonstrates a well-defined, compressible fluid collection, which has low echogenicity and contains septa. Haemoglobin breakdown products produce a characteristic T2 hypointense signal on MRI [105]. This effect is magnified by gradient-echo sequences, on which a ‘blooming’ artefact can be seen. The T1 and T2 signal intensities of haematomas evolve over times in line with evolving haemoglobin breakdown products. One should be cautious when diagnosing haematomas, because haematomas and soft tissue sarcomas can be difficult to differentiate clinically and radiologically. There are several case reports of sarcomas being misdiagnosed as haematomas, most commonly when the patient has a history of mild trauma.

**Fig. 34:**
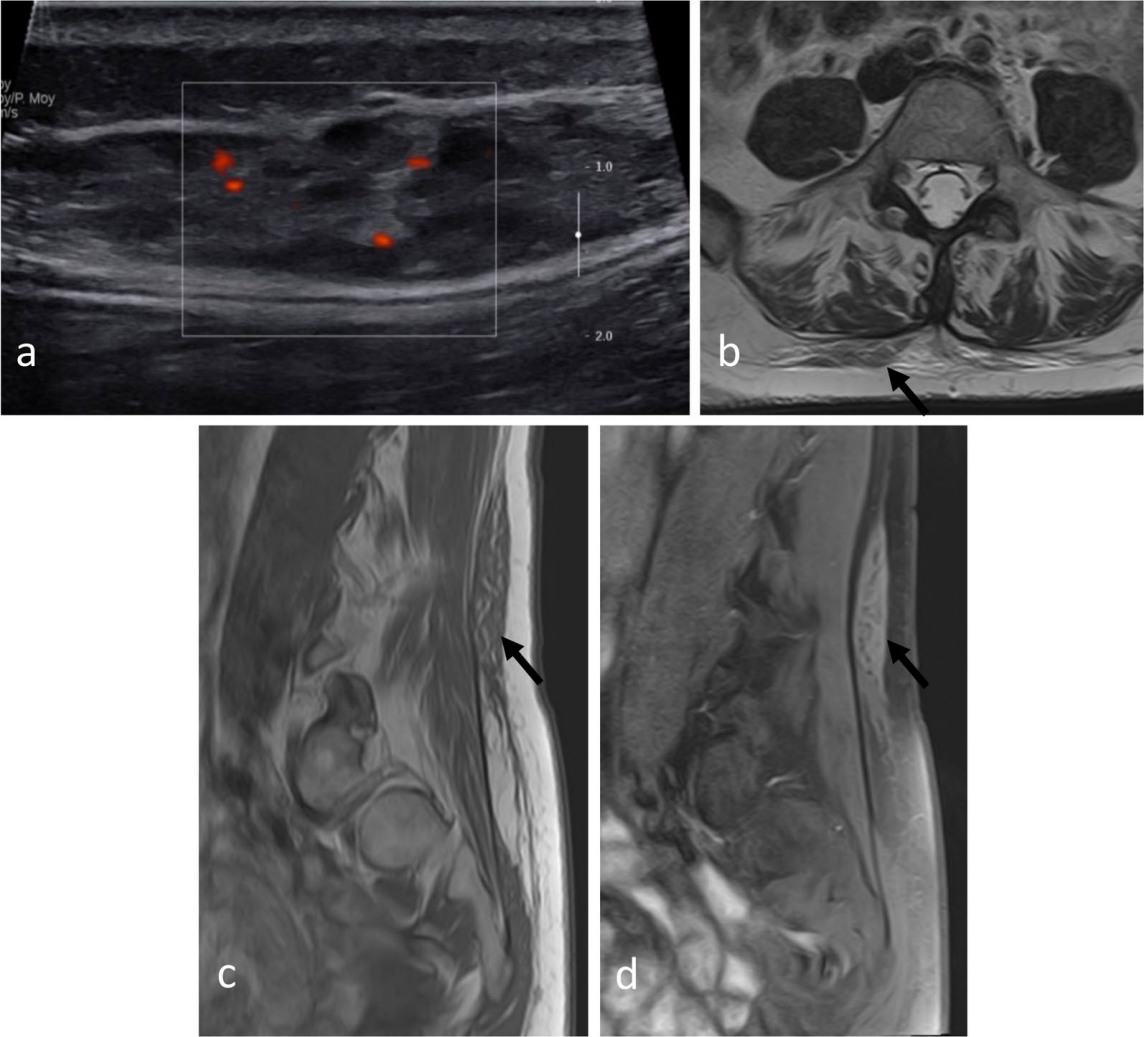
*Adiponecrosis* in a 43-year-old male with low back pain 2 months after a fall. US (**a**) demonstrating a hyperechoic, superficial mass and power Doppler signal showing hypervascularity. Axial T2WI (**b**) and sagittal T1WI (**c**) showing a superficial mass (arrows) with heterogeneous iso- to hyperintense signal. Sagittal contrast-enhanced fat-suppressed T1WI (**d**) showing a heterogeneous enhancement

The radiological appearance of *fat necrosis* is a subcutaneous, ill-defined mass with fibrous tissue with hypointensity on T2WI intermixed with adipose tissue with hyperintensity on T1WI (Fig. 34) [106]. Peripheral enhancement is frequent. Given the severity of differential diagnoses, which can include liposarcomas, histological proof is needed to confirm a diagnosis. *Morel Lavallée* results from traumatic shearing force with separation of the subcutaneous fat from the underlying fascia and occurs most frequently in road traffic accident [107]. The back is the second most common location after the thigh.

### Degenerative disorders of the spine

Spinal degeneration is a complex entity involving vertebral bodies, intervertebral discs and facet joints, as well as spinous processes and flaval and interspinous ligaments. Spinal degenerative disorders may be associated with small size abnormalities in the paraspinal soft tissues, including lumbar interspinous *bursitis*, which is shown as a fluid-like signal located between adjacent pathological spinous processes, *cystic formations* of the mobile spine or *fibrous nodules* on the side of the spinous processes or lysis (Additional file 1: Fig. S3) [108].

### Calcifications and ossifications

*Myositis ossificans* (MOC) is non-neoplastic abnormal extraosseous bone formation [109]. MOC occurs usually in young adults and can be either traumatic, which is the most common, or non-traumatic in origin. They are extremely rare in the paraspinal muscles, in the largest reported case series, only one of the 68 patients was found in the paraspinal muscles. The radiological appearance of MOC evolves as the lesion matures. At an early stage, MRI shows an ill-defined muscle mass with a central high signal intensity and no adjacent bone abnormalities. After a few weeks, a peripheral rim of mineralisation appears around the lesion, with a periosteal reaction also present in the adjacent bone.

*Spinal crystal deposition* may cause paraspinal pseudomasses, particularly in the setting of an acute inflammatory presentation [110]. Paraspinal abnormalities are usually adjacent to erosive facet joints, or less frequently to erosive vertebral plates. Tophi or calcium pyrophosphate deposition appears hypo- or isointense compared with muscles on both T1WI and T2WI with peripheral enhancement. Crystal deposition can be seen on CT images in the form of a dense mass.

### Infection

*Spinal infections, discitis–osteomyelitis (DOM)* and *septic facet joints* may affect the paraspinal region in many ways: reactive inflammation, pyomyositis and abscesses can occur in the epaxial muscles around the affected joint (Fig. 11) [111]. DOM is often associated with paraspinal soft tissue abnormalities, especially in hypaxial muscles, close to the anterior endplates, where DOM originates from. In the early stages of DOM, high T2 signal intensity in the psoas musculature, known as the ‘imaging psoas sign’, is suggestive of DOM [112]. Paraspinal abscesses occur as a late presentation of spinal infection. Well-defined paraspinal abnormal enhancement and thin and smooth rim enhancement of paraspinal abscesses and calcifications within abscesses are suggestive of tuberculous DOM [113]. In cases of tuberculosis, large paraspinal abscesses, termed ‘cold abscesses’, can develop without severe pain or prominent inflammatory signs and symptoms.

### Extramedullary haematopoiesis

**Fig. 35:**
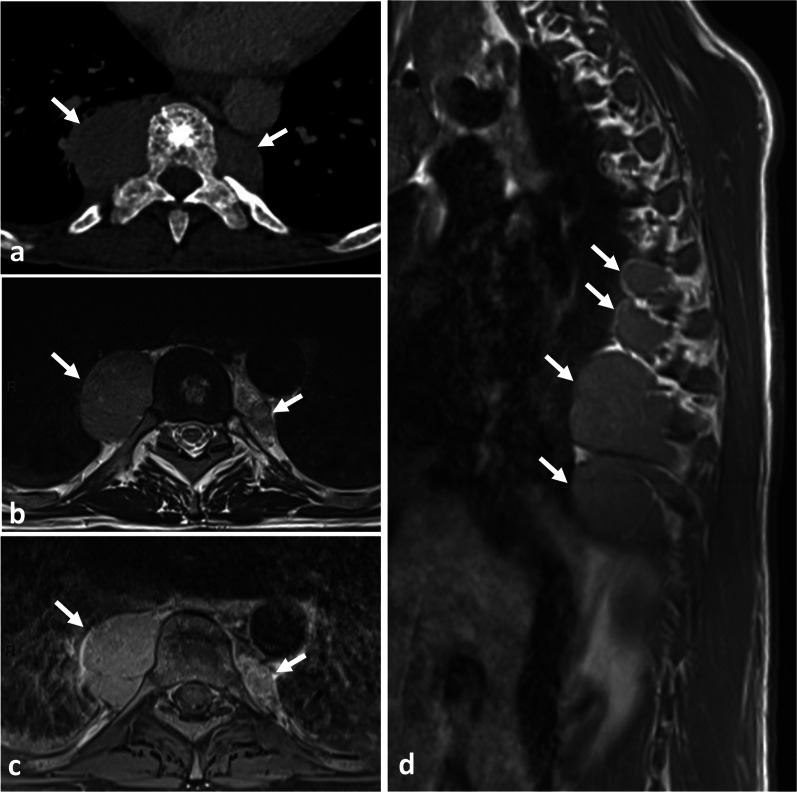
*Extramedullary haematopoiesis* in a 25-year-old male with sickle cell disease. Contrast-enhanced axial CT scan reconstruction in the bone window (**a**), axial T2WI (**b**) and axial contrast-enhanced T1WI (**c**) show well-defined, bilateral hypaxial masses (arrows). Sagittal T1WI demonstrates multiple hypaxial masses (arrows) (**d**). Axial CT scan reconstruction (**a**) shows widened ribs and osteosclerosis of the vertebra

Extramedullary haematopoiesis is an expansion of haematopoietic tissue outside the bone marrow medulla. EMH can be caused by many haematological diseases, including myeloproliferative neoplasms or chronic haematological and genetic disorders such as thalassemia or sickle cell disease. EMH commonly occurs in the paraspinal region (also epidural) and is multifocal and/or bilateral [114]. On CT and MR imaging, EMH presents as smoothly lobulated paravertebral masses. Recent active masses are homogeneous, with CT attenuation similar to that of the adjacent muscle, with hypointensity on T1WI and hyperintensity on T2WI (Fig. 35). Older inactive masses appear heterogeneous, especially in contrast-enhanced CT and MRI scans, due to iron deposition and fat infiltration. EMH is frequently associated with radiological skeletal changes related to chronic anaemia, including widened ribs, trabeculation and cortical thinning.

## Multidisciplinary team management and surgical considerations

As a rule, it is recommended that patients with paraspinal masses are directed to referral centres with expertise in their management. The management strategy must be individualised and coordinated by a MDT consisting of radiologists, oncologists, pathologists, surgeons and so forth.

Although not all paraspinal masses require surgical treatment, clinicians should always ask whether this therapeutic option should be considered. For most benign and malignant aggressive neoplasms, *en bloc* resection is favoured. An economic resection along the pseudocapsule is the rule for benign tumours. For malignant tumours, the tumour’s margins must be considered; thus, excision should leave a layer of healthy tissue around the tumour. Occasionally for soft tissue tumours, this implies a periosteal resection and/or bony resection. Hence, surgical treatment of paraspinal and spinal tumours may be challenging owing to their proximity to important structures such as the spinal cord, nerve roots, great vessels and chest. Therefore, any surgical indication should be weighed against the consequences of sacrificing these structures if required. In fact, MDT discussions often centre around tumour resectability and imaging analysis.

Neoadjuvant radiotherapy and/or chemotherapy may be administered to reduce the volume of some tumours, thus reducing the extent of resection required and maximising the chances of obtaining a resection with tumour-free margins.

Commonly, tumours with vertebral invasion require a vertebrectomy, which can be partial or total. For thoracic spinal tumours, a preoperative angiography is obtained to localise the Adamkiewicz’s artery. Vascularised tumours are embolised to decrease operative blood loss. For tumours invading the spine or originating from the spine, the surgical procedure involves a vertebral resection and nerve root sacrifice followed by spinal reconstruction with grafts and implants [40]. Supplemental resection, such as chest wall, pleura, pulmonary lobe resection, may be required for thoracic tumours extending out of the paraspinal mass. The most important radiological parameters for selecting the type of vertebrectomy include invasion of transverse processes, vertebral foramina, vertebral bodies and the epidural space.

## Conclusion

Paraspinal masses can be challenging for every practitioner involved in oncology MDTs (i.e. radiologists, oncologists, pathologists and surgeons). Overall, there are three main nosological entities: soft tissue tumours, spinal tumours and non-neoplastic pseudomasses. Characterisation of the vascular, neural and bone extension of a paraspinal tumour is crucial to determine suitable patient management. MRI is the imaging modality of choice to accurately describe the locoregional extension of the tumour; however, combining several imaging modalities is almost always required to assess the mineralisation and vascularisation of the mass, the bone involvement and the visibility of the lesion on a radiograph. Clinicians should be aware that the proportion of malignant versus benign tumours in the paraspinal region is greater than that of tumours of the limbs. Suspicious masses should raise concern and lead to referral to centres with expertise in their diagnosis and treatment.

## Supplementary Information

**Additional file 1: Supplementary fig 1**. Solitary fibrous tumour of the paraspinal region in a 45-year-old male. Axial T2WI (a) shows a paraspinal soft-tissue tumour with an extension into the spinal canal via the intervertebral foramen (white arrows) and intercostal space (arrowhead). The tumour extends along three vertebral levels as shown on sagittal T2WI (b) and sagittal contrast-enhanced fat suppressed T1WI.** Supplementary fig 2**. Plexiform neurofibromas in a 27-year-old male with neurofibromatosis type 1. Axial T2WI (a) and post-contrast T1WI (b) show infiltrative diffuse subcutaneous neurofibromas (arrowheads) and plexiform neurofibromas (arrows) in psoas muscles.** Supplementary fig 3**. Cystic formation of the mobile spine at L5-S1 level in a 68-year-old-male. Sagittal T2WI (a), sagittal T1WI (b) and axial T2 (c) show a small juxtafact cyst with fluid-like intensity on all MR sequences. Note the fatty degeneration and atrophy of the right multifidus on axial T2WI (c).

## Data Availability

Not applicable.
