# Supplementary material for: Understanding a mass in the paraspinal region: an anatomical approach
Source: Insights Imaging. 2023 Jul 19;14:128. doi: 10.1186/s13244-023-01462-1 (PMC10356722; doi:10.1186/s13244-023-01462-1)

Understanding a mass in the paraspinal region: An anatomical approach

ELECTRONIC SUPPLEMENTARY MATERIAL

Supplementary Figure 1:

Solitary fibrous tumour of the paraspinal region in a 45-year-old male with non-mechanical back pain. Axial T2WI (a) shows a paraspinal soft-tissue tumour with an extension into the spinal canal via the intervertebral foramen (white arrows) and intercostal space (arrowhead). The tumour extends along three vertebral levels as shown on sagittal T2WI (b) and sagittal contrast-enhanced fat suppressed T1WI.

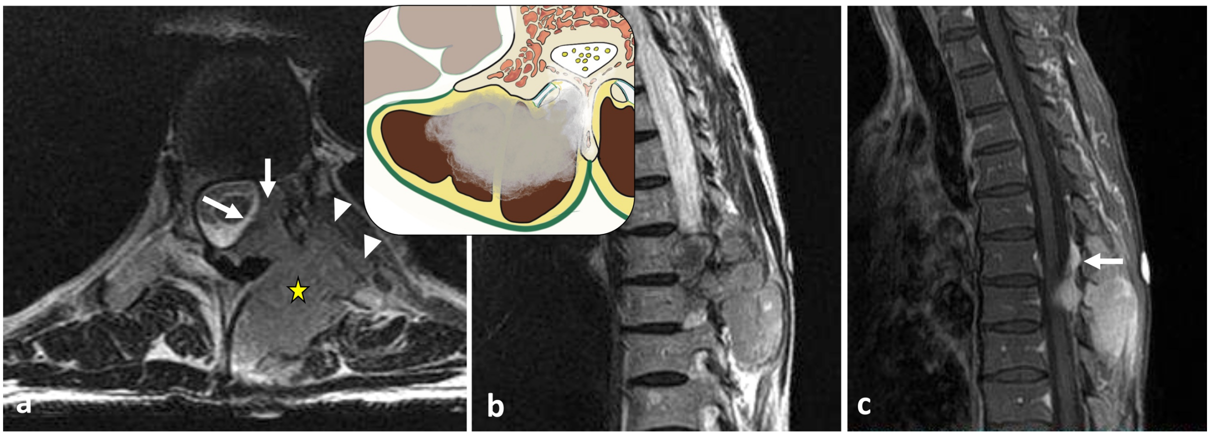

Supplementary Figure 2:

Plexiform neurofibromas in a 27-year-old male with neurofibromatosis type 1. Axial T2WI (a) and post-contrast T1WI (b) show infiltrative diffuse subcutaneous neurofibromas (arrowheads) and plexiform neurofibromas (arrows) in psoas muscles.

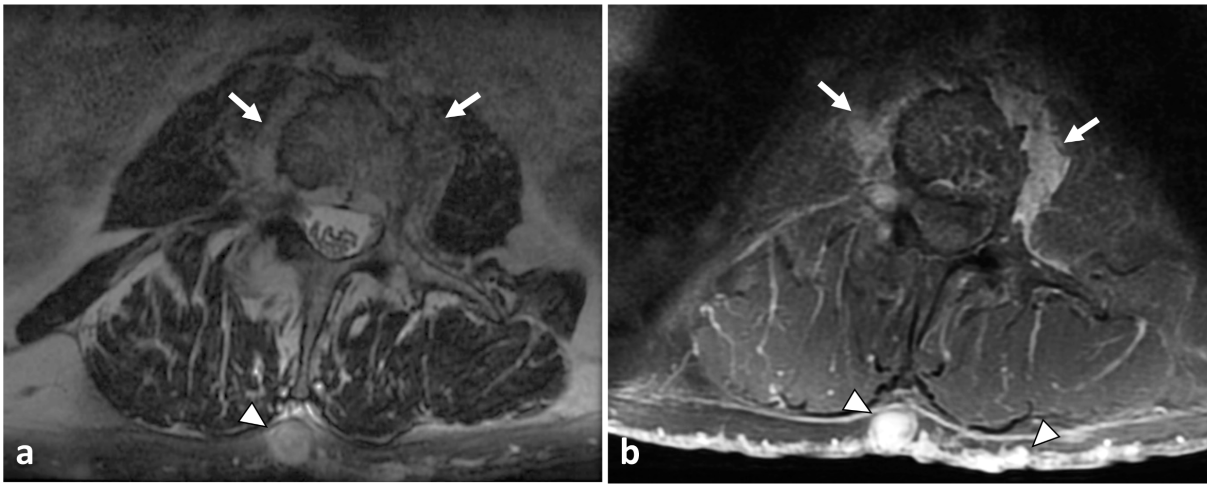

Supplementary Figure 3:

Cystic formation of the mobile spine at L5-S1 level in a 68-year-old-male. Sagittal T2WI (a), sagittal T1WI (b) and axial T2 (c) show a small juxtafacet cyst with fluid-like intensity on all MR sequences. Note the fatty degeneration and atrophy of the right multifidus on axial T2WI (c).

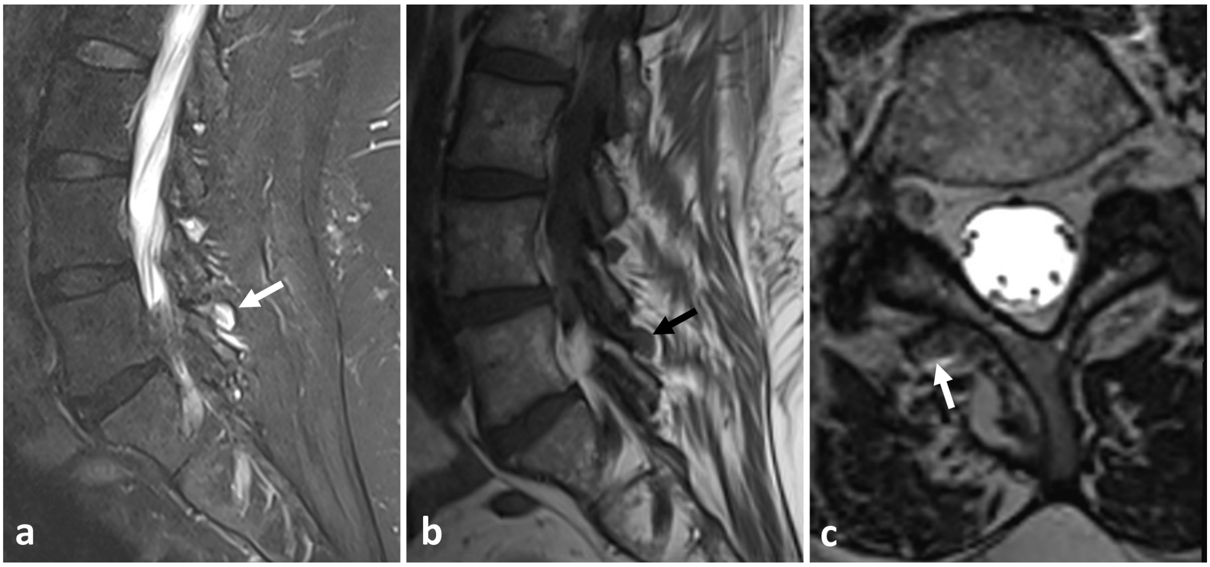

Supplement: Supplementary file 1 — Additional file 1: Supplementary fig 1. Solitary fibrous tumour of the paraspinal region in a 45-year-old male. Axial T2WI (a) shows a paraspinal soft-tissue tumour with an extension into the spinal canal via the intervertebral foramen (white arrows) and intercostal space (arrowhead). The tumour extends along three vertebral levels as shown on sagittal T2WI (b) and sagittal contrast-enhanced fat suppressed T1WI. Supplementary fig 2. Plexiform neurofibromas in a 27-year-old male with neurofibromatosis type 1. Axial T2WI (a) and post-contrast T1WI (b) show infiltrative diffuse subcutaneous neurofibromas (arrowheads) and plexiform neurofibromas (arrows) in psoas muscles. Supplementary fig 3. Cystic formation of the mobile spine at L5-S1 level in a 68-year-old-male. Sagittal T2WI (a), sagittal T1WI (b) and axial T2 (c) show a small juxtafact cyst with fluid-like intensity on all MR sequences. Note the fatty degeneration and atrophy of the right multifidus on axial T2WI (c). [file 13244_2023_1462_MOESM1_ESM.pdf]
